# Pharmacokinetic comparison of quercetin, isoquercitrin, and quercetin-3-O-β-D-glucuronide in rats by HPLC-MS

**DOI:** 10.7717/peerj.6665

**Published:** 2019-03-26

**Authors:** Hongli Yin, Ji Ma, Jichun Han, Maoru Li, Jing Shang

**Affiliations:** 1 State Key Laboratory of Natural Medicines, China Pharmaceutical University, Nanjing, China; 2 School of Traditional Chinese Pharmacy, China Pharmaceutical University, Nanjing, China; 3 Jiangsu Key Laboratory of TCM Evaluation and Translational Research, China Pharmaceutical University, Nanjing, China

**Keywords:** Pharmacokinetic, Biotransformation, Quercetin, Isoquercitrin, Quercetin-3-O-β-D-glucuronide

## Abstract

**Background:**

Quercetin (Qr), isoquercitrin (IQ), and quercetin-3-O-β-D-glucuronide (QG) are powerful phytochemicals that have been shown to exhibit disease prevention and health promotion properties. However, there may exist transformations between Qr, IQ, and QG *in vivo*. And the pharmacokinetic profiles of Qr, IQ, and QG have not been systematically compared. The pharmacokinetics study would be helpful to better understand the pharmacological actions of them.

**Methods:**

Herein, we developed a reliable HPLC-MS method to compare the pharmacokinetics of Qr, IQ, and QG after separate (50 mg/kg) oral administration of them in rats, using puerarin as internal standard. The detection was performed using negative selected ion monitoring. This method was validated in terms of selectivity, linearity, precision, accuracy, extraction recovery, matrix effect, and stability; and shows reliabilities in monitoring the pharmacokinetic behaviors of these three compounds.

**Results:**

Our results showed that after separate oral administration of Qr, IQ, and QG, all of the compounds could be detected in plasma. In addition, QG could be detected in the Qr group; Qr and QG could be measured in the IQ group; and Qr could be found in rat plasma after 1.5 h of QG administration. Moreover, the AUC_0−*t*_ of Qr in the; Qr group (2,590.5 ± 987.9 mg/L*min), IQ group (2,212.7 ± 914.1 mg/L*min), and QG group (3,505.7 ± 1,565.0 mg/L*min) was larger than the AUC_0−*t*_ of QG in the; Qr group (1,550.0 ± 454.2 mg/L*min), IQ group (669.3 ± 188.3 mg/L*min), and QG group (962.7 ± 602.3 mg/L*min). The AUC_0−*t*_ of IQ was the lowest among all groups.

**Discussion:**

Quercetin, IQ, and QG can all be absorbed into plasma. A mutual transformation exists between Qr and QG, and IQ can be metabolized into Qr and QG in SD rats. These results would provide a meaningful basis for understanding the pharmacological actions of these three compounds.

## Introduction

Flavonols with the quercetin structure are found in many medicinal plants (Sun, Simonyi & Sun, 2002) and have attracted significant attention as powerful phytochemicals for disease prevention and health promotion (Fraga, Celep & Galleano, 2009; Murakami, Ashida & Terao, 2008). Quercetin (Qr), isoquercitrin (IQ), and quercetin-3-O-β-D-glucuronide (QG), representatives of quercetin flavonols, are present in many herbal plants, such as *Cichorium glundulosum Boiss et Hout* (Ding et al., 2014), *Nelumbo nucifera* (Kitanov, 1988), and *Hypericum hirsutum* (Kashiwada et al., 2005). Qr is one of the most widely used dietary flavonoids and its anti-atherosclerotic, lipid-modulating, and anti-carcinogenic effects have been extensively studied (Aguirre et al., 2014; Terao, Kawai & Murota, 2008; Youdim, Shukitt-Hale & Joseph, 2004). IQ, the glycoside form of Qr, has attracted increasing attention owing to its activity against cardiovascular disorders, diabetes, cancer, and allergic reactions (Valentová et al., 2014; Vrba et al., 2013). Many studies (Cermak, Landgraf & Wolffram, 2003; Terao, 2009; Terao, Murota & Kawai, 2011) have proposed that QG—the glucuronide form of Qr—functions not only as a detoxified metabolite also as a hydrophobic aglycone precursor and hydrophilic bioactive agent for various ROS-generating systems of quercetin aglycone. Compared with the long history of application and widespread research on Qr, the research on IQ and QG is much less extensive.

Pharmacokinetic studies of drugs are necessary for understanding their *in vivo* behavior and pharmacological mechanisms. Quercetin flavonols are usually administered orally and can undergo biotransformation by intestinal microorganisms and metabolism in the liver (Day et al., 2003; Murota et al., 2000; Petri et al., 2003; Spencer et al., 1999; Walle, 2004). As a result, the main metabolites in plasma may be the bioactive components. Compared with the parent compounds under different conditions, metabolites may produce similar, stronger or even weaker effects (Crespy, 1998; Day et al., 2000a; Sesink, O’Leary & Hollman, 2001). In addition, since most research on the pharmacokinetic characteristics of Qr, IQ, and QG uses crude herbal extracts, other components of the extracts may affect their pharmacokinetic characteristics, and even contribute contrasting results (Paganga & Rice-Evans, 1997; Sesink, O’Leary & Hollman, 2001). It has been reported that a mutual transformation between Qr and QG may exist *in vivo* (Guo et al., 2014; Menendez et al., 2011; Yang et al., 2016), and a small number of studies have compared the metabolism of IQ and Qr (Paulke et al., 2012). However, after the separate oral administration of Qr, IQ, and QG in rats, their pharmacokinetics and biotransformation the compounds remain unclear. To address this, in this study we established a sensitive and reliable HPLC-MS method for simultaneous determination of Qr, IQ, and QG in rat plasma and subsequently used the method to systematically compare the pharmacokinetic characteristics of Qr, IQ, and QG in rats. It was expected that the results of this study would help to explain the metabolic relationships between the compounds and could be applied in pharmacological studies for better understanding of the pharmacological effects of each compound.

## Materials and Methods

### Chemicals and materials

**Figure 1:**
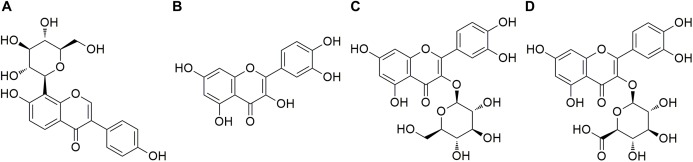
Chemical structures of the analytes and IS. (A) Puerarin (IS), (B) Quercetin (Qr), (C) Isoquercitrin (IQ), (D) Quercetin-3-O-β-D-glucuronide (QG).

Quercetin (purity ≥ 98%, CAS: 117-39-5) and isoquercitrin (purity ≥ 98%, CAS: 21637-25-2) were provided by the Aladdin Reagent Co. Ltd. (Shanghai, China). Puerarin (purity ≥ 98%, CAS: 3681-99-0) as the internal standard (IS) was purchased from the National Institutes for Food and Drug Control (Beijing, China). Quercetin-3-O-β-D-glucuronide (purity ≥ 96%, CAS: 22688-79-5) was obtained from Sigma-Aldrich (St. Louis, MO, USA). Their structures are shown in Fig. 1. HPLC grade formic acid, acetonitrile, and methanol were provided by Merck (Darmstadt, Germany), and ultrapure water (18 MΩ) was prepared in our laboratory using Milli-Q system (Millipore, Burlington, MA, USA). Other chemicals and reagents were of analytical grade.

### Pharmacokinetics study

A total of 15 SPF Sprague-Dawley rats (SD, male, 190–210 g) were provided by Sino-British Sippr/BK Lab Animal Company Ltd. (Shanghai, China). The rats were kept in standard conditions (22–26 °C; 40–60% relative humidity; 12 h light/dark cycle). Throughout the rearing period, the animals had access to food and water ad libitum. Animal experiments were conducted in accordance with the standard ethical guidelines of the Laboratory Animal Management Committee of Jiangsu Province (SYXK (SU) 2016-0011).

Following 1 week of adaptation, the rats were fasted for about 12 h before dosing, with free access to water. A total of 15 rats were randomly divided into Qr, IQ, and QG groups (*n* = 5), and were dosed accordingly by oral gavage (50 mg/kg/day). After administration of the first dose, blood samples were taken from the retro-orbital vein at 0, 5, 15, 30, 60, 90, 120, 180, 240, 360, 480, and 1,440 min and collected in heparinized tubes. The samples were centrifuged at 3,500 rpm for 10 min at 4 °C and the plasma was stored at −80 °C until analysis.

### Instrumentation and HPLC-MS method

An Agilent 1,200 series HPLC system (Agilent Technologies, Waldbronn, Germany) equipped with a quaternary pump on-line degasser, auto-sampler and column oven, and an SL G1946D quadruple mass spectrometer (Agilent Technologies, Santa Clara, CA, USA) equipped with an electrospray ionization (ESI) source, were used for separation and detection. Data were acquired and analyzed using Agilent ChemStation Software Version A.01.00 (Agilent Technologies, Santa Clara, CA, USA).

The separation was carried out with an Amethyst C18-H column (150 × 4.6 mm, 5 μm, Sepax Technologies, Newark, DE, USA) with a guard column SB-C18 (10 × 4.6 mm, 5 μm, Phenomenex Technologies, Torrance, USA) at a constant temperature of 25 °C. The mobile phase was composed of aqueous solution (0.4% formic acid, A) and acetonitrile (B) and the program of the mobile phase was as follows: 0 min, 5% B; 5 min, 12% B; 10 min, 33% B; 15 min, 50% B; 18 min, 60% B, at a flow rate of 0.8 mL/min with 5 μL sample injection volume.

The mass spectra were acquired using ESI interfaced to a single quadrupole mass spectrometer. The ionization conditions were as follows: drying gas (N_2_) flow rate of 11 L/min, drying gas temperature of 350 °C, nebulizing gas (N_2_) pressure of 35 psi, capillary voltage of 3,500 V, quad temperature of 100 °C, and fragment voltage of 110 V. A series of m/z were monitored using a timed program according to the retention times and characteristic ions for the corresponding HPLC-MS peak obtained by scanning from m/z 100–1,000 in the standard sample. The analytes were monitored using negative selected ion monitoring (SIM) at m/z 301.1 for Qr [M-H]^−^, m/z 463.1 for IQ [M-H]^−^, m/z 477.1 for QG [M-H]^−^, and m/z 415.2 for IS [M-H]^−^.

### Preparation of standards and quality control samples

The stock solutions of Qr, IQ, and QG were separately prepared by dissolving the reference standards in methanol at a final concentration of 0.210, 0.192, and 0.200 mg/mL, respectively. The standard working solutions were prepared by diluting with methanol to the desired concentrations. The calibration curves of Qr, IQ, and QG were in the range of 32.8–10,500.0, 24.4–976.0, and 50.0–12,500.0 ng/mL, respectively. The QC samples at low, medium and high concentrations were prepared in the same way and the concentrations at three levels were as follows: 65.6, 525.0, 8,400.0 ng/mL for Qr, 48.8, 244.0, 976.0 ng/mL for IQ, and 100.0, 1,000.0, 10,000.0 ng/mL for QG, respectively. An IS stock solution (0.192 mg/mL) was prepared by dissolving the puerarin standard in methanol and stored at 4 °C.

### Sample preparation

Briefly, an aliquot of plasma sample (100 μL) was spiked with 5% formic acid (10 μL) in centrifuge tube. Subsequently, hydrochloric acid (2 mol/L, 10 μL) and IS solution (960.00 ng/mL, 20 μL) were added. Then ethyl acetate/n-butyl alcohol (1:1 v/v; 1,000 μL) was used to extract the components. The samples were then homogenized by vortex mixing and centrifuged for 10 min at 14,000 rpm. After centrifugation, 800 μL of supernatant was transferred to another centrifuge tube and the sample was evaporated to dryness. The residues were dissolved in 100 μL of methanol and centrifuged for 10 min at 12,000 rpm. 50 μL of the supernatant was transferred to the vial and an aliquot of 5 μL was injected into the HPLC-MS system for analysis.

### Method validation

The HPLC-MS method used in this study was verified for specificity, linearity, precision, accuracy, extraction recovery, matrix effect, and stability (Food and Drug Administration of the United States, 2013).

#### Specificity

The specificity was assessed by comparing six independent individual blanks, spiked and drug-administered plasma samples to check for the presence of any potential interference in the peak region of the analytes and IS using the proposed extraction procedure and analytical conditions.

#### Linearity and lower limits of quantification

A best-fit calibration curve was produced by plotting the area ratios (*y*-axis) of the analytes to IS against the concentrations of Qr, IQ, and QG (*x*-axis) in the form of *y* = *bx* + *a*, which was then evaluated using least-squares linear regression analysis weighted (1/*x*^2^). The Linearity and lower limits of quantification (LLOQs) was defined as the lowest concentrations of the calibration curve with accuracy (relative error, RE) and precision (relative standard deviation, RSD) not exceeding ± 20%.

#### Precision and accuracy

The intra- and inter-day precision and accuracy of the method were assessed by replicate analysis (*n* = 6) of QC samples at low, medium and high concentrations on the same day (intra-day) and three consecutive days (inter-day). Accuracy and precision at each QC concentration were expressed as RE% and RSD%, respectively.

#### Extraction recovery and matrix effect

To evaluate extraction recoveries, the first set of QC samples (*n* = 6) were prepared by spiking three flavonoids into blank plasma at three different concentration levels. Then the samples were processed using the procedure described above. The second set of blank plasma samples were extracted and spiked the QC samples. Extraction recovery values for analytes were determined by calculating the ratios of the raw peak areas of the QC samples with those of spike-after-extraction samples. The matrix effect was evaluated by comparing the responses of the analytes at three different levels in the post-extraction spiked blank plasma with those of the corresponding standard solutions.

#### Stability

The stabilities of Qr, IQ, and QG in plasma were assessed by analyzing QC samples at different storage conditions. The stabilities of three flavonoids in plasma were studied by analyzing three concentration levels of QC samples (*n* = 5) at room temperature (25 °C) for 12 h, at long-term storage (−80 °C for 15 days), after three freeze (−80 °C) and thaw (room temperature) cycles, and at auto sampler rack storage (4 °C for 24 h).

### Data analysis

DAS 2.0 (Chinese Pharmacological Association, Shanghai, China) was used to calculate the pharmacokinetic parameters using the non-compartment model, including the area under curve (AUC), mean residence time (MRT), half-life ( *t_1/2z_*), clearance rate (CL), maximum plasma concentration (*C*_max_), and time (*T*_max_). All results are expressed as mean ± standard deviation (SD). One way analysis of variance followed by Kruskal–Wallis test was used for comparison of different groups using Graph pad prism 5 software. A *p*-value of <0.05 was considered statistically significant.

## Results

### Optimization of HPLC-MS conditions

Under the optimized ESI conditions, a comparison of the responses to Qr, IQ, QG, and IS in positive and negative ion HPLC-MS modes revealed higher responses and lower noise in negative mode. An Amethyst C18-H column was used to separate Qr, IQ, QG, and IS with high efficiency. To achieve good chromatographic behavior, formic acid, acetic acid, and ammonium acetate aqueous solution were investigated. We found that 0.4% formic acid aqueous presented better method sensitivity and could significantly enhance the signal response of all analytes.

### Method validation

#### Specificity

**Figure 2:**
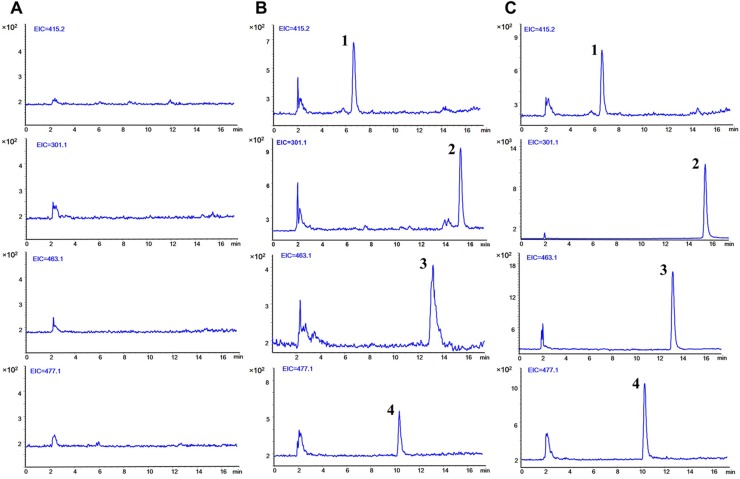
Typical selected ion monitoring (SIM) chromatograms of (1) IS, (2) quercetin, (3) isoquercitrin, and (4) quercetin 3-O-β-D-glucuronide in rat plasma. (A) blank plasma. (B) blank plasma spiked with the three analytes at lower limit of quantification (LLOQ) and IS. (C) after 5 min oral administration of quercetin (50 mg/kg), isoquercitrin (50 mg/kg), quercetin-3-O-β-D-glucuronide (50 mg/kg) seperately to rats and IS. (1, puerarin, 6.76 min; 2, quercetin, 15.56 min; 3, isoquercitrin, 13.29 min; 4, quercetin-3-O-β-D-glucuronide, 10.53 min).

The typical SIM chromatograms of the blank plasma, blank plasma spiked with three analytes at LLOQ level and IS, and plasma obtained 5 min after separate oral administration of Qr, IQ, and QG are presented in Fig. 2. No interference was observed at the respective times of the analytes and IS. The retention time was 15.56 min for Qr, 13.29 min for IQ, 10.53 min for QG, and 6.76 min for IS.

#### Calibration curves and LLOQs

The calibration curves and the linear range for the analytes are shown in Table 1. The correlation coefficients (*r*) for every calibration curves exceeded 0.99, showing good linearity over the concentration range. The accuracy and precision of LLOQs were within ± 20%, which would be sufficient for pharmacokinetic study of three flavonoids.

**Table 1:** The regression equations, linear range and lower limit of quantification (LLOQ) of the analytes.

Biosamples	Compounds	Regression equation	Correlation coefficient (*r*)	Linear range (ng/mL)	LLOQ (ng/mL)
Plasma	Quercetin (Qr)	*y* = 0.002550x−0.031	0.995	32.8–10,500.0	32.8
	Isoquercitrin (IQ)	*y* = 0.001785x + 0.132	0.998	24.4–976.0	24.4
	Quercetin-3-O-β-D-glucuronide (QG)	*y* = 0.001876x + 0.208	0.996	50.0–12,500.0	50.0

#### Precision and accuracy

The precision and accuracy were evaluated by determining three levels of QC samples, and the results are summarized in Table S1. The precision ranged from 5.6% to 14.6%, and the accuracy values ranged from −13.8% to 6.4%. These results indicated that the present HPLC-MS method was reliable, accurate and reproducible.

#### Matrix effect and recovery

The mean extraction recoveries of Qr, IQ, and QG at all QC concentration levels in plasma ranged from 68.9% to 79.2% (*n* = 6, Table S2). The extraction recoveries of analytes in plasma were stable. The matrix effects for the analytes in plasma ranged from 96.8% to 106.5% at each QC level indicative of a lack of apparent endogenous matrix (Table S2).

#### Stability

The stabilities of Qr, IQ, and QG in plasma were assessed by analyzing three concentration levels of QC samples at different storage conditions (Table S3). The results showed that these analytes in plasma sample were all stable at room temperature for 12 h, at −80 °C for 15 days, and after three freeze-thaw cycles. Post-preparation stability of the analytes also showed no significant degradation when the extracted samples were kept in the auto sampler rack at 4 °C for 24 h.

### Pharmacokinetics study

**Figure 3:**
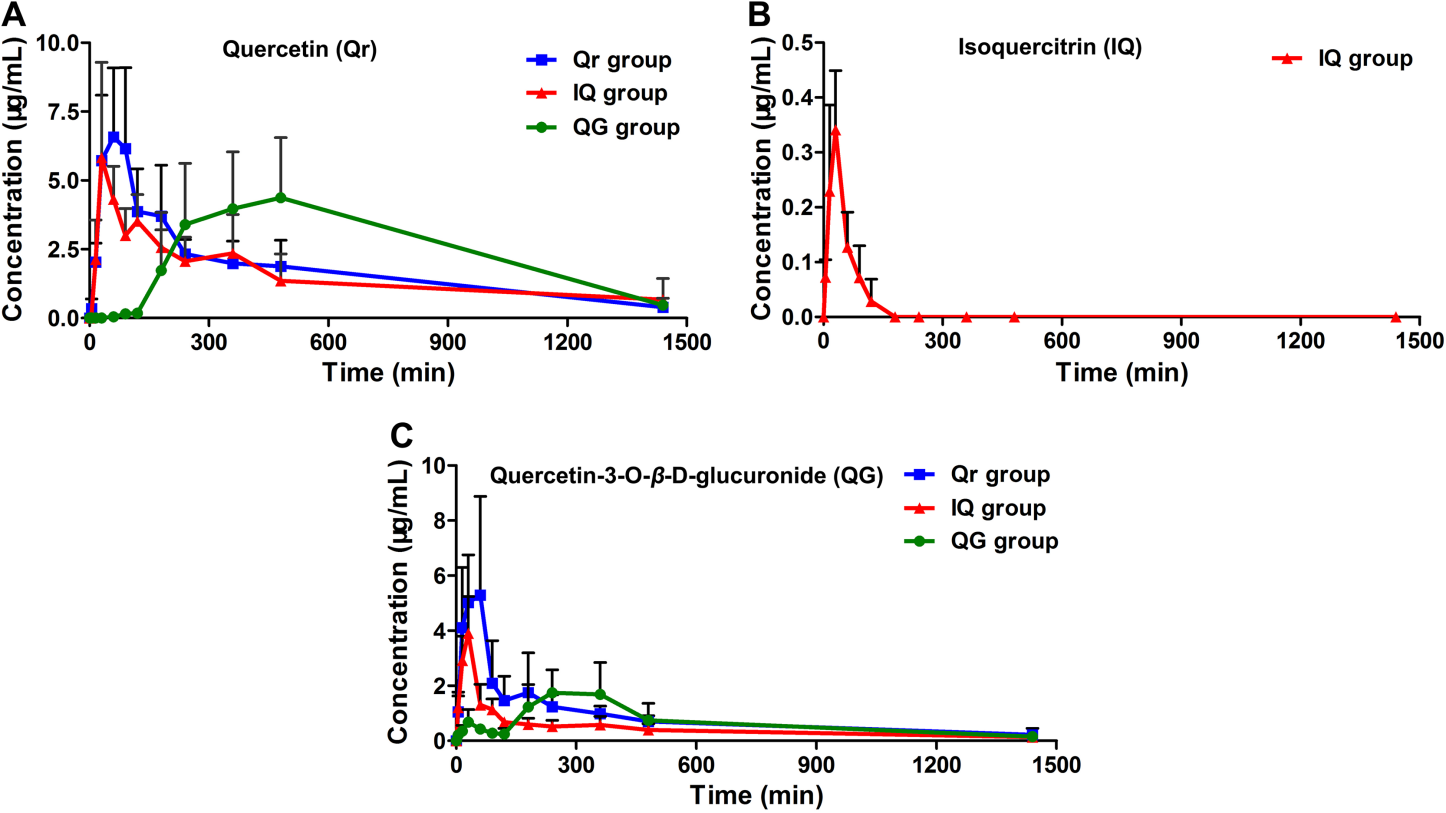
Mean plasma concentration-time profiles of (A) quercetin (Qr), (B) isoquercitrin (IQ), and (C) quercetin-3-O-β-D-glucuronide (QG) after oral administration of 50 mg/kg Qr, IQ, and QG separately in rats. (Mean ± SD, *n* = 5).

The developed HPLC-MS method was applied to study the pharmacokinetics of Qr, IQ, and QG in rats. The mean plasma concentration-time profiles for Qr, IQ, and QG after oral administration in rats are presented in Fig. 3. The pharmacokinetic parameters are summarized in Table 2. After Qr (50 mg/kg) was orally administrated to rats, Qr could be detected in rat plasma and reached the *C*_max_ (7.47 ± 2.63 ug/mL) at *T*_max_ (54.0 ± 25.1 min) with the AUC_0−*t*_ (2,590.5 ± 987.9 mg/L*min) (Table 2). After IQ (50 mg/kg) was orally administrated to rats, IQ could be detected in rat plasma and reached the *C*_max_ (0.35 ± 0.11 ug/mL) at *T*_max_ (27.0 ± 6.7 min) with the AUC_0−*t*_ (17.2 ± 7.3 mg/L*min) (Table 2). Moreover, after QG (50 mg/kg) was orally administrated to rats, QG could be detected in rat plasma and reached the *C*_max_ (2.04 ± 0.85 ug/mL) at *T*_max_ (222.0 ± 119.2 min) with the AUC_0−*t*_ (962.7 ± 602.3 mg/L*min) (Table 2). The AUC of IQ could only be accessed until 90–120 min, unlike the AUC of others groups. Greater absorption was observed for Qr and QG than for IQ (Table 2). *T*_max_ (222.0 ± 119.2 min) of QG was delayed compared with *T*_max_ (54.0 ± 25.1 min) of Qr (Table 2). In addition, a clear double peak phenomenon was observed in QG mean plasma concentration-time profile after oral administration of QG (Fig. 3C).

**Table 2:** Pharmacokinetic parameters of quercetin (Qr), isoquercitrin (IQ), and quercetin-3-O-β-D-glucuronide (QG) after oral administration of 50 mg/kg Qr, IQ, and QG separately in rats. (Mean ± SD, *n* = 5).

Pharmacokinetic parameters	Qr group		IQ group			QG group	
	Qr	QG	IQ	Qr	QG	QG	Qr
AUC_0−*t*_ (mg/L*min)	2,590.5 ± 987.9	1,150.0 ± 454.2	17.2 ± 7.3	2,212.7 ± 914.1	669.3 ± 188.3	962.7 ± 602.3	3,505.7 ± 1,565.0
MRT_0−*t*_ (min)	383.3 ± 24.3	315.9 ± 134.7	40.6 ± 7.2	422.6 ± 148.6	355.9 ± 94.6	437.8 ± 36.3	491.1 ± 22.6
*t*_1/2z_ (min)	437.3 ± 54.3	545.8 ± 436.6	42.6 ± 27.9	378.4 ± 338.8	523.5 ± 190.4	376.4 ± 95.2	365.4 ± 152.1
*T*_max_ (min)	54.0 ± 25.1	42.0 ± 16.4	27.0 ± 6.7	42.0 ± 16.4	30.0 ± 0.0	222.0 ± 119.2	360 ± 120
CL (L/min/kg)	0.02 ± 0.008	0.043 ± 0.029	2.91 ± 1.29	0.024 ± 0.011	0.07 ± 0.02	0.071 ± 0.057	0.014 ± 0.014
*C*_max_ (ug/mL)	7.47 ± 2.63	6.46 ± 2.94	0.35 ± 0.11	6.60 ± 2.67	3.91 ± 1.34	2.04 ± 0.85	5.17 ± 1.85

**Note:**

AUC, the area under concentration-time curve; MRT, mean residence time; *t_1/2z_*, half-life; *C*_max_, maximum plasma concentration; and *T*_max_, time; CL, clearance rate.

**Figure 4:**
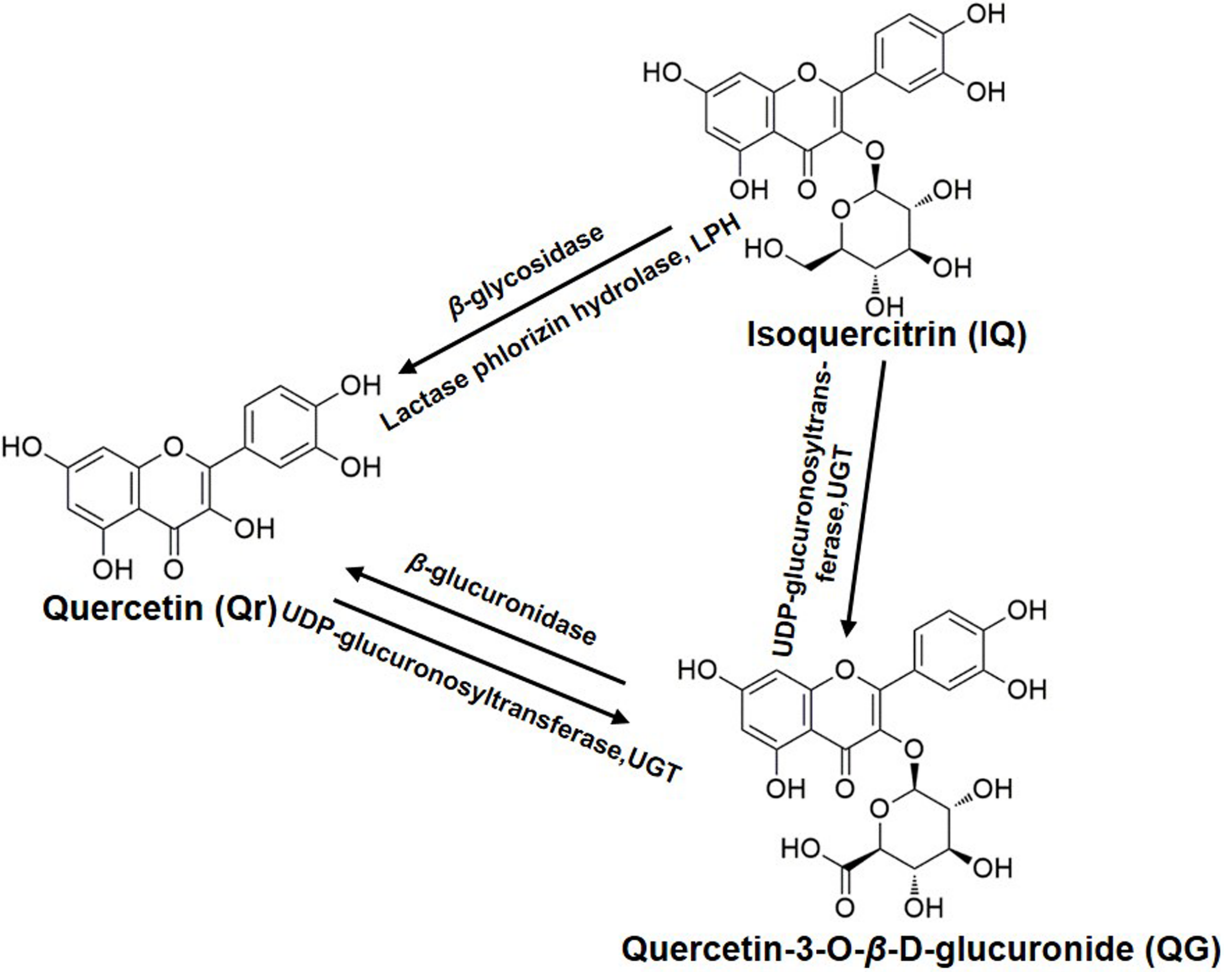
The biotransformation among quercetin, isoquercitrin, and quercetin-3-O-β-D-glucuronide.

Furthermore, QG was detected in the Qr group (Fig. 3C); Qr and QG were measured in the IQ group (Figs. 3A and 3C); And Qr was found in rat plasma after 1.5 h of QG administration (Fig. 3A). The potential biotransformation relationship is summarized in Fig. 4. In addition, the AUC_0−*t*_ of Qr in the Qr group (2,590.5 ± 987.9 mg/L*min), IQ group (2,212.7 ± 914.1 mg/L*min), and QG group (3,505.7 ± 1,565.0 mg/L*min) was larger than the AUC_0−*t*_ of QG in the Qr group (1,150.0 ± 454.2 mg/L*min), IQ group (669.3 ± 188.3 mg/L*min), and QG group (962.7 ± 602.3 mg/L*min) (Table 2). The MRT_0−*t*_ of Qr in all groups was also larger than that of QG in the corresponding group, and the MRT_0−*t*_ of IQ was the lowest among all groups. The *T*_max_ of Qr in the QG group was significantly delayed compared with that of Qr in the Qr (*p* = 0.02) and IQ groups (*p* = 0.006). The *T*_max_ of QG in the QG group also came after a longer period than that of QG in the Qr and IQ (*p* = 0.021) groups. And the *T*_max_, *t*_1/2z_, and *C*_max_ of IQ were the shortest and lowest compared with Qr and QG. The CL of IQ was the fastest compared with Qr and QG (Table 2).

## Discussion

Our results indicated that Qr, IQ, and QG can all be absorbed into plasma; There is a biotransformation between Qr and QG; And IQ can be metabolized into Qr and QG. Under the same intragastric dosage (50 mg/kg) conditions, the AUC values for Qr in the Qr, IQ, and QG dose groups were larger than those for QG in the corresponding groups. And the AUC of IQ was the lowest. In addition, each pharmacokinetic profile (Fig. 3) came from different individual rats, there existed transformations between Qr, IQ, and QG *in vivo*, and the metabolism and transformation between these three compounds would be affect by many factors, such as gastric emptying rate and intestinal flora (Rowland et al., 2018; Yuan et al., 2016). The above reasons might leaded to the large data variability of Qr, IQ, or QG used for calculating the mean pharmacokinetic profiles and the phenomenon of large data variability also could be found in previous study (Yang et al., 2016).

In previous studies, Sesink, O’Leary & Hollman (2001) and Murota et al. (2010) revealed that after administration of IQ in human subjects, in contrast to QG, Qr and IQ were not detected in plasma. In contrast to these reports, some recent studies have found that after administration of IQ-enriched herbal extracts, intact IQ can be detected in rat plasma (He et al., 2013; Li et al., 2010; Xue et al., 2011; Zhou et al., 2011). These reports all used effective plasma sample extraction and sensitive HPLC or LC/MS analysis methods, however, the metabolism of IQ could be affected by the complex mixtures of herbal extracts (Zuo et al., 2006). Studies by Paulke et al. (2012) and Lu et al. (2013) found that after oral administration of IQ, Qr could be detected in plasma, which agrees with our findings (Fig. 3A). Chang et al. (2005) found that after a single oral dose of IQ (4.5 mg/kg) in rats, quercetin glucuronide and a trace amount of IQ could be found in plasma, which is consistent with our results (Figs. 3C and 3B). Valentová et al. (2014) proposed that although small amounts of intact IQ can be found in plasma after oral administration, IQ still shows many chemical protective effects both *in vitro* and *in vivo*.

Quercetin is weakly acidic and can be absorbed in the stomach (Crespy et al., 2002). Unlike Qr, IQ cannot be absorbed (Crespy et al., 2002) and is stable in the stomach (Chang et al., 2005; Zuo et al., 2006). However, IQ can undergo deglycosylation by lactase phlorizin hydrolase present on the outer surface of the intestinal brush border, or by microbial enzymes (Schoefer et al., 2003) to give Qr (Day et al., 2000b). If absorbed intact, IQ can be hydrolyzed to Qr in intestinal cells by cytosolic β-glycosidase (Lesser, Cermak & Wolffram, 2004). These are potential reasons why Qr can be detected in plasma after oral administration of IQ (Fig. 4).

The biotransformation of Qr and IQ can also take place in the liver. LC-MS results showed that the predominant biotransformation processes for Qr and its glycosides were methylation and glucuronidation, produced by isolated human or rat hepatocytes (Vacek et al., 2012); which could account for QG being detected in the blood after IQ or Qr were administrated (Fig. 3C). Studies suggest that Qr can be glucuronidated by UDP-glucuronyltransferases, which are present in human and rat intestine (Cermak, Landgraf & Wolffram, 2003; Lesser, Cermak & Wolffram, 2004) (Fig. 4). Yang et al. (2016) found that QG circulates in the bloodstream after oral administration of Qr. As the hydrophobic glucuronide form of Qr cannot easily cross cell membranes by diffusion, its absorption and bioavailability are hampered. This may be one of the reasons for the delayed *T*_max_ of QG compared with that of Qr (Table 2).

Generally, flavonoid conjugates are hydrolyzed to aglycone before passing through the small intestine, while flavonoid aglycone can be directly absorbed into epithelial cells in the intestine (Kroon et al., 2004; Manach et al., 1997). Spencer et al. (1999) found that the hydrolysate of IQ and glucuronide in isolated rat intestine could release Qr. We also found that after IQ or QG were administrated, Qr could be detected in the blood (Fig. 3A). Yang et al. (2016) demonstrated that double peaks are observed after oral administration of QG in rats, which is consistent with our findings (Fig. 3C). Previous work indicates that delayed gastric emptying, enterohepatic recirculation, and variability of absorption may cause double-peaked pharmacokinetics (Huh, 2007; Lee & Mitchell, 2012; Xing, Chen & Zhong, 2005).

Furthermore, QG was found to be much less toxic compared with its aglycone (Yoshino et al., 2011). The lower toxicity of QG appears to be derived from its extremely high hydrophilicity. In addition, the lack of a free OH group at the three-position contributes to the less toxic effect of QG (Awad et al., 2000; Boots, Haenen & Bast, 2008). QG has been shown to be the more effective anti-oxidative metabolite in rat plasma after oral administration of Qr (Moon et al., 2001; Shirai et al., 2002), and it (10 mM) can inhibit lipid peroxidation (Shirai et al., 2001). It is likely that QG can release Qr by enhanced β-glucuronidase activity during inflammation (Kawai et al., 2008; Shimoi et al., 2001) (Fig. 4).

The results obtained in this study corroborate with the literature and show with pharmacokinetic profiles that there exist biotransformation between Qr and QG after administration of the compounds separately. Also, these results confirm that Qr and QG are metabolites of IQ. The pharmacokinetic profiles obtained after administration of analytical standards help to explain the relation between the compounds. These results can be applied to pharmacokinetic/pharmacodynamic studies for better understanding of the pharmacological effects of each compound.

## Conclusions

A sensitive HPLC-MS method was successfully developed, validated, and used to determine the pharmacokinetic profiles of Qr, IQ, and QG. Using liquid–liquid extraction with a mixture of n-butyl alcohol and ethyl acetate to simultaneously analyze complex mixtures of Qr, IQ, and QG with a range of low polarity to high polarity compounds, was more effective than protein precipitation (methanol and acetonitrile). Furthermore, we compared the pharmacokinetics of Qr, IQ, and QG and found that there is biotransformation between Qr and QG, and IQ can be metabolized into Qr and QG. The pharmacokinetic profiles obtained after administration of analytical standards help to explain the relation between the compounds and is helpful for understanding their pharmacodynamics mechanism. In addition, the results provide a guidance for their clinical use.

## Supplemental Information

10.7717/peerj.6665/supp-1 Supplemental Information 1 Precision and accuracy of the three analytes in rat plasma. Given data show quality control samples at low, medium and high concentrations on the same day (intra-day) and three consecutive days (inter-day). Accuracy and precision at each quality control concentration were expressed as RE% and RSD%, respectively. RE = relative error; RSD = relative standard deviation.

10.7717/peerj.6665/supp-2 Supplemental Information 2 Extract recoveries and matrix effects of the three analytes in rat plasma (*n* = 6). Given data show the extract recoveries and matrix effects of quercetin (Qr), isoquercitrin (IQ), and quercetin-3-O-β-D-glucuronide (QG) in rat plasma. RSD = relative standard deviation.

10.7717/peerj.6665/supp-3 Supplemental Information 3 Stability of the three analytes in rat plasma (*n* = 5). Given data show that the stabilities of quercetin (Qr), isoquercitrin (IQ), and quercetin-3-O-β-D-glucuronide (QG) in plasma were studied by analyzing three concentration levels of quality control samples (*n* = 5) at room temperature (25 °C) for 12 h, at long-term storage (−80 °C for 15 days), after three freeze (−80 °C) and thaw (room temperature) cycles, and at auto sampler rack storage (4 °C for 24 h). RSD = relative standard deviation.

10.7717/peerj.6665/supp-4 Supplemental Information 4 Raw data exported from the ChemDraw software applied for preparation for Figs. 1 and 4 for the chemical structures of puerarin (IS), quercetin (Qr), isoquercitrin (IQ), and quercetin-3-O-β-D-glucuronide (QG).

10.7717/peerj.6665/supp-5 Supplemental Information 5 Raw data exported from the Agilent ChemStation Software applied for preparation for Fig. 2 for the selected ion monitoring chromatograms of puerarin (IS), quercetin (Qr), isoquercitrin (IQ), and quercetin-3-O-β-D-glucuronide (QG). Raw data exported from the Agilent ChemStation Software applied for preparation for Fig. 2 for the selected ion monitoring chromatograms of puerarin (IS), quercetin (Qr), isoquercitrin (IQ), and quercetin-3-O-β-D-glucuronide (QG).

10.7717/peerj.6665/supp-6 Supplemental Information 6 Raw data exported from the Graph pad prism 5 software applied for preparation for Fig. 3 for the mean plasma concentration-time profiles of quercetin (Qr), isoquercitrin (IQ), and quercetin-3-O-β-D-glucuronide (QG).

10.7717/peerj.6665/supp-7 Supplemental Information 7 Raw data exported from the DAS 2.0 software applied for data analyses and preparation for Table 1 for the standard regression equations of quercetin (Qr), isoquercitrin (IQ), and quercetin-3-O-β-D-glucuronide (QG).

10.7717/peerj.6665/supp-8 Supplemental Information 8 Raw data exported from the DAS 2.0 software applied for data analyses and preparation for Table 2 for the pharmacokinetic parameters of quercetin (Qr), isoquercitrin (IQ), and quercetin-3-O-β-D-glucuronide (QG). The area under concentration-time cur.

10.7717/peerj.6665/supp-9 Supplemental Information 9 Raw data applied for data analyses and preparation for Table S1 for the precision and accuracy of quercetin (Qr), isoquercitrin (IQ), and quercetin-3-O-β-D-glucuronide (QG).

10.7717/peerj.6665/supp-10 Supplemental Information 10 Raw data applied for data analyses and preparation for Table S2 for the extraction recoveries and matrix effects of quercetin (Qr), isoquercitrin (IQ), and quercetin-3-O-β-D-glucuronide (QG).

10.7717/peerj.6665/supp-11 Supplemental Information 11 Raw data applied for data analyses and preparation for Table S3 for the stability of quercetin (Qr), isoquercitrin (IQ), and quercetin-3-O-β-D-glucuronide (QG).

## Additional Information and Declarations

### Data Availability

The following information was supplied regarding data availability: The raw measurements are available in the Supplemental Files.
