# Supplementary material for: Pharmacokinetic comparison of quercetin, isoquercitrin, and quercetin-3-O-β-D-glucuronide in rats by HPLC-MS
Source: PeerJ. 2019 Mar 26;7:e6665. doi: 10.7717/peerj.6665 (PMC6440464; doi:10.7717/peerj.6665)
Supplement: Supplemental Information 1 — Given data show quality control samples at low, medium and high concentrations on the same day (intra-day) and three consecutive days (inter-day). Accuracy and precision at each quality control concentration were expressed as RE% and RSD%, respectively. RE = relative error; RSD = relative standard deviation. [file peerj-07-6665-s001.docx]

**Table S1.** Precision and accuracy of the three analytes in rat plasma.

| Compounds | Nominal Conc. (ng/mL) | Intra-day (n=6) | | | Inter-day (n=18, 3 days) | | |
| --- | --- | --- | --- | --- | --- | --- | --- |
|  |  | Measured Conc. (ng/mL) | RSD (%) | RE (%) | Measured Conc. (ng/mL) | RSD (%) | RE (%) |
| Quercetin (Qr) | 65.6 | 60.9±8.9 | 14.6 | -7.2 | 62.3±7.7 | 12.4 | -5.1 |
|  | 525.0 | 558.7±48.4 | 8.7 | 6.4 | 539.4±48.2 | 8.9 | 2.7 |
|  | 8400.0 | 8568.7±623.4 | 7.3 | 2.0 | 8651.0±483.9 | 5.6 | 3.0 |
| Isoquercitrin (IQ) | 48.8 | 42.1±3.6 | 8.6 | -13.8 | 44.6±4.6 | 10.2 | -8.6 |
|  | 244.0 | 254.8±29.3 | 11.5 | 4.4 | 258.3±32.4 | 12.6 | 5.9 |
|  | 976.0 | 955.6±114.3 | 12.0 | -2.1 | 950.8±100.1 | 10.5 | -2.6 |
| Quercetin-3-O-*β*-D-glucuronide (QG) | 100.0 | 103.1±10.1 | 9.8 | 3.1 | 92.8±9.0 | 9.7 | -7.2 |
|  | 1000.0 | 1017.8±114.9 | 11.3 | 1.8 | 955.0±88.6 | 9.3 | -4.5 |
|  | 10000.0 | 10211.2±1348.9 | 13.2 | 2.1 | 9740.3±1125.1 | 11.6 | -2.6 |
