# Supplementary material for: Pharmacokinetic comparison of quercetin, isoquercitrin, and quercetin-3-O-β-D-glucuronide in rats by HPLC-MS
Source: PeerJ. 2019 Mar 26;7:e6665. doi: 10.7717/peerj.6665 (PMC6440464; doi:10.7717/peerj.6665)
Supplement: Supplemental Information 2 — Given data show the extract recoveries and matrix effects of quercetin (Qr), isoquercitrin (IQ), and quercetin-3-O-β-D-glucuronide (QG) in rat plasma. RSD = relative standard deviation. [file peerj-07-6665-s002.docx]

**Table S2.** Extract recoveries and matrix effects of the three analytes in rat plasma (n = 6).

| Compounds | Nominal Conc. (ng/mL) | Extract recovery (%) | RSD (%) | Matrix effect (%) | RSD (%) |
| --- | --- | --- | --- | --- | --- |
| Quercetin (Qr) | 65.6 | 79.2±3.5 | 4.4 | 96.8±6.5 | 6.7 |
|  | 525.0 | 71.9±6.6 | 9.2 | 101.4±9.3 | 9.2 |
|  | 8400.0 | 73.3±7.6 | 10.3 | 104.7±5.6 | 5.3 |
| Isoquercitrin (IQ) | 48.8 | 76.9±6.3 | 8.2 | 103.5±7.5 | 7.2 |
|  | 244.0 | 75.2±7.4 | 9.8 | 106.5±5.2 | 4.8 |
|  | 976.0 | 68.9±8.8 | 12.7 | 101.4±5.6 | 5.5 |
| Quercetin-3-O-*β*-D-glucuronide (QG) | 100.0 | 70.2±5.0 | 7.1 | 101.2±10.9 | 10.8 |
|  | 1000.0 | 73.5±5.7 | 7.8 | 103.5±8.8 | 8.5 |
|  | 10000.0 | 69.5±9.6 | 13.8 | 104.1±10.1 | 9.7 |
