# Supplementary material for: Pharmacokinetic comparison of quercetin, isoquercitrin, and quercetin-3-O-β-D-glucuronide in rats by HPLC-MS
Source: PeerJ. 2019 Mar 26;7:e6665. doi: 10.7717/peerj.6665 (PMC6440464; doi:10.7717/peerj.6665)
Supplement: Supplemental Information 3 — Given data show that the stabilities of quercetin (Qr), isoquercitrin (IQ), and quercetin-3-O-β-D-glucuronide (QG) in plasma were studied by analyzing three concentration levels of quality control samples (n = 5) at room temperature (25 °C) for 12 h, at long-term storage (−80 °C for 15 days), after three freeze (−80 °C) and thaw (room temperature) cycles, and at auto sampler rack storage (4 °C for 24 h). RSD = relative standard deviation. [file peerj-07-6665-s003.docx]

**Table S3.** Stability of the three analytes in rat plasma (n=5).

| Compounds | Nominal Conc. (ng/mL) | Short-term stability (25℃ for 12 h) | | Long-term stability (-80℃ for 15 d) | | Free-thaw stability (3 free-thaw cycles) | | Post-preparation stability (4℃ for 24 h) | |
| --- | --- | --- | --- | --- | --- | --- | --- | --- | --- |
|  |  | Measured Conc. (ng/mL) | RSD (%) | Measured Conc. (ng/mL) | RSD (%) | Measured Conc. (ng/mL) | RSD (%) | Measured Conc. (ng/mL) | RSD (%) |
| Quercetin (Qr) | 65.6 | 60.8±3.5 | 5.7 | 60.0±4.7 | 7.8 | 65.3±6.9 | 10.5 | 61.0±4.5 | 7.4 |
|  | 525.0 | 474.0±37.4 | 7.9 | 500.7±36.0 | 7.2 | 511.9±40.6 | 7.9 | 526.3±60.5 | 11.5 |
|  | 8400.0 | 7949.4±241.7 | 3.0 | 7982.9±795.7 | 10.0 | 8631.3±801.3 | 9.3 | 7642.7±675.6 | 8.8 |
| Isoquercitrin (IQ) | 48.8 | 51.3±1.6 | 3.0 | 43.5±2.7 | 6.3 | 50.4±1.8 | 3.6 | 45.3±1.4 | 3.1 |
|  | 244.0 | 229.0±14.0 | 6.1 | 227.0±9.1 | 4.0 | 242.5±20.7 | 8.5 | 255.6±16.0 | 6.2 |
|  | 976.0 | 902.7±36.8 | 4.1 | 947.8±75.3 | 8.0 | 1029.0±62.9 | 6.1 | 937.6±55.4 | 5.9 |
| Quercetin-3-O-β-D-glucuronide (QG) | 100.0 | 89.3±6.5 | 7.2 | 98.6±10.2 | 10.3 | 98.4±7.0 | 7.1 | 97.2±6.0 | 6.2 |
|  | 1000.0 | 943.2±64.9 | 6.9 | 941.5±102.3 | 10.9 | 954.9±93.5 | 9.8 | 910.7±68.0 | 7.5 |
|  | 10000.0 | 9334.6±320.2 | 3.4 | 9985.0±971.4 | 9.7 | 9353.2±620.8 | 6.6 | 9409.6±569.5 | 6.1 |
