# Supplementary figures and images for: Pharmacokinetic comparison of quercetin, isoquercitrin, and quercetin-3-O-β-D-glucuronide in rats by HPLC-MS
Source: PeerJ. 2019 Mar 26;7:e6665. doi: 10.7717/peerj.6665 (PMC6440464; doi:10.7717/peerj.6665)

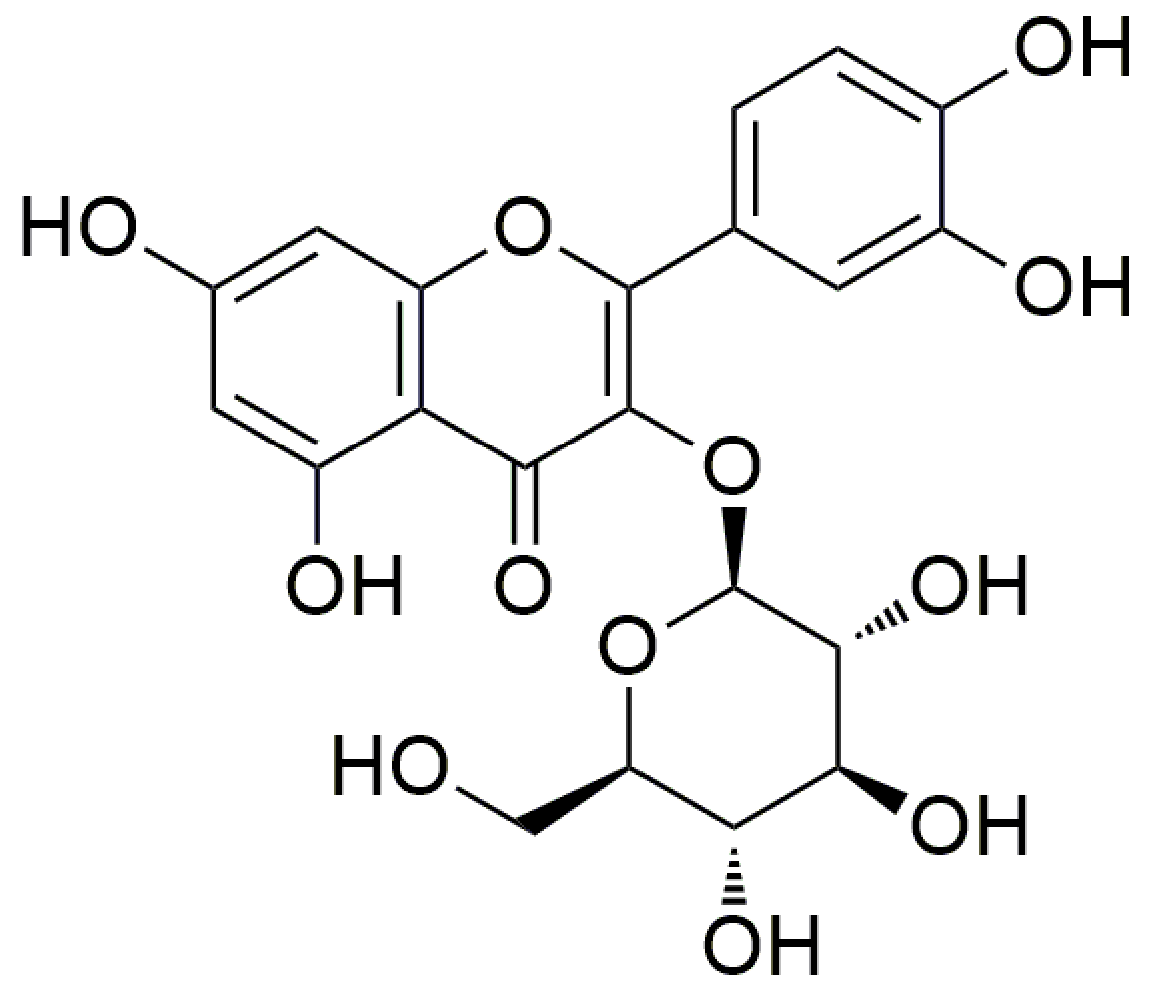

Supplement: Supplemental Information 4 [file peerj-07-6665-s004.zip › Supplemental_Data_S1_a/Isoquercitrin (IQ).png]

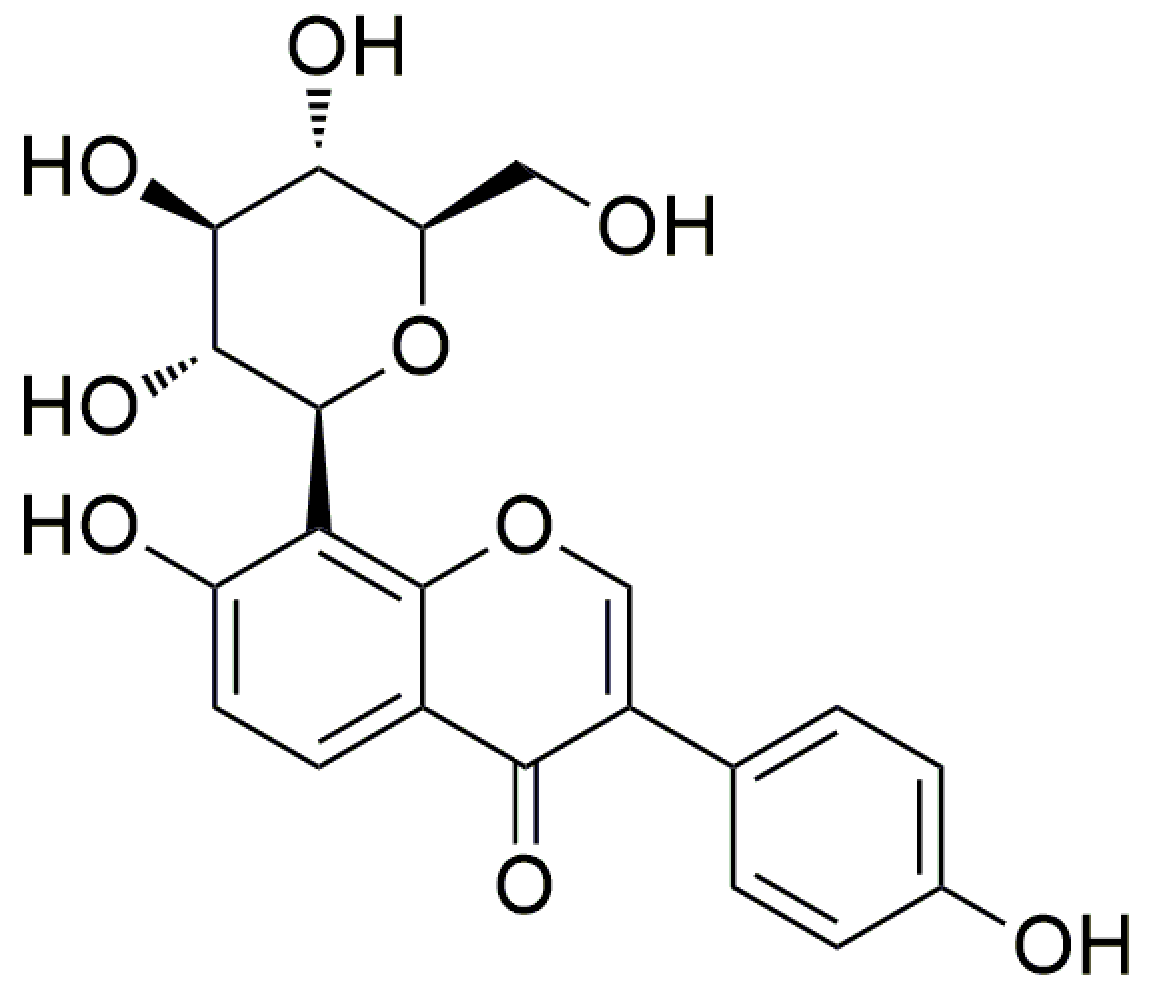

Supplement: Supplemental Information 4 [file peerj-07-6665-s004.zip › Supplemental_Data_S1_a/Puerarin (IS).png]

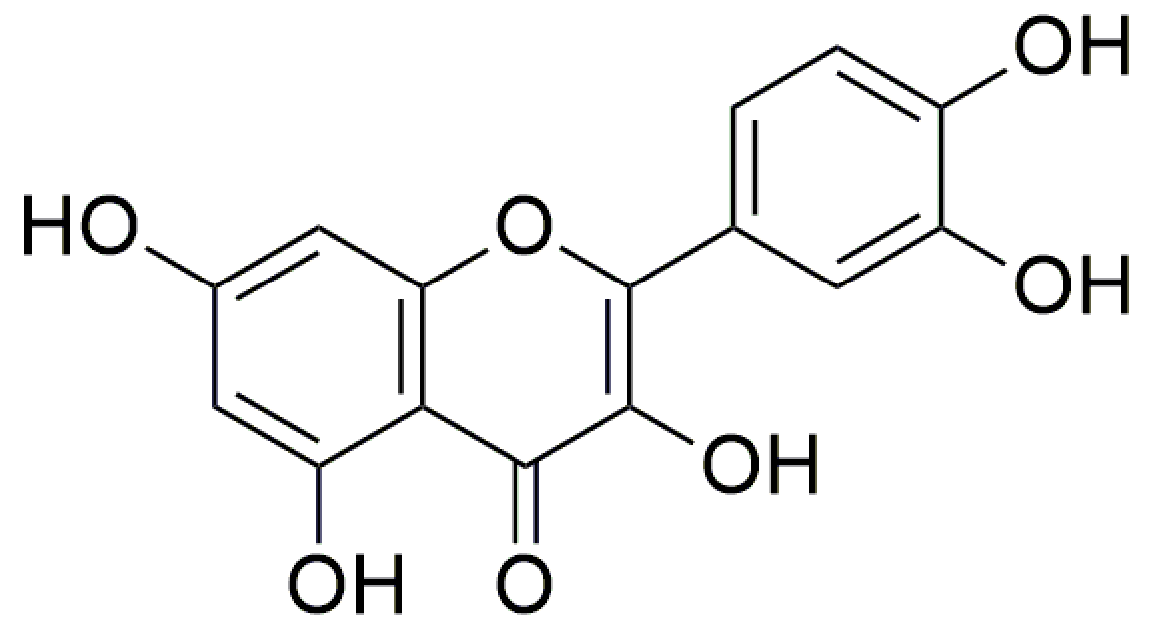

Supplement: Supplemental Information 4 [file peerj-07-6665-s004.zip › Supplemental_Data_S1_a/Quercetin (Qr).png]

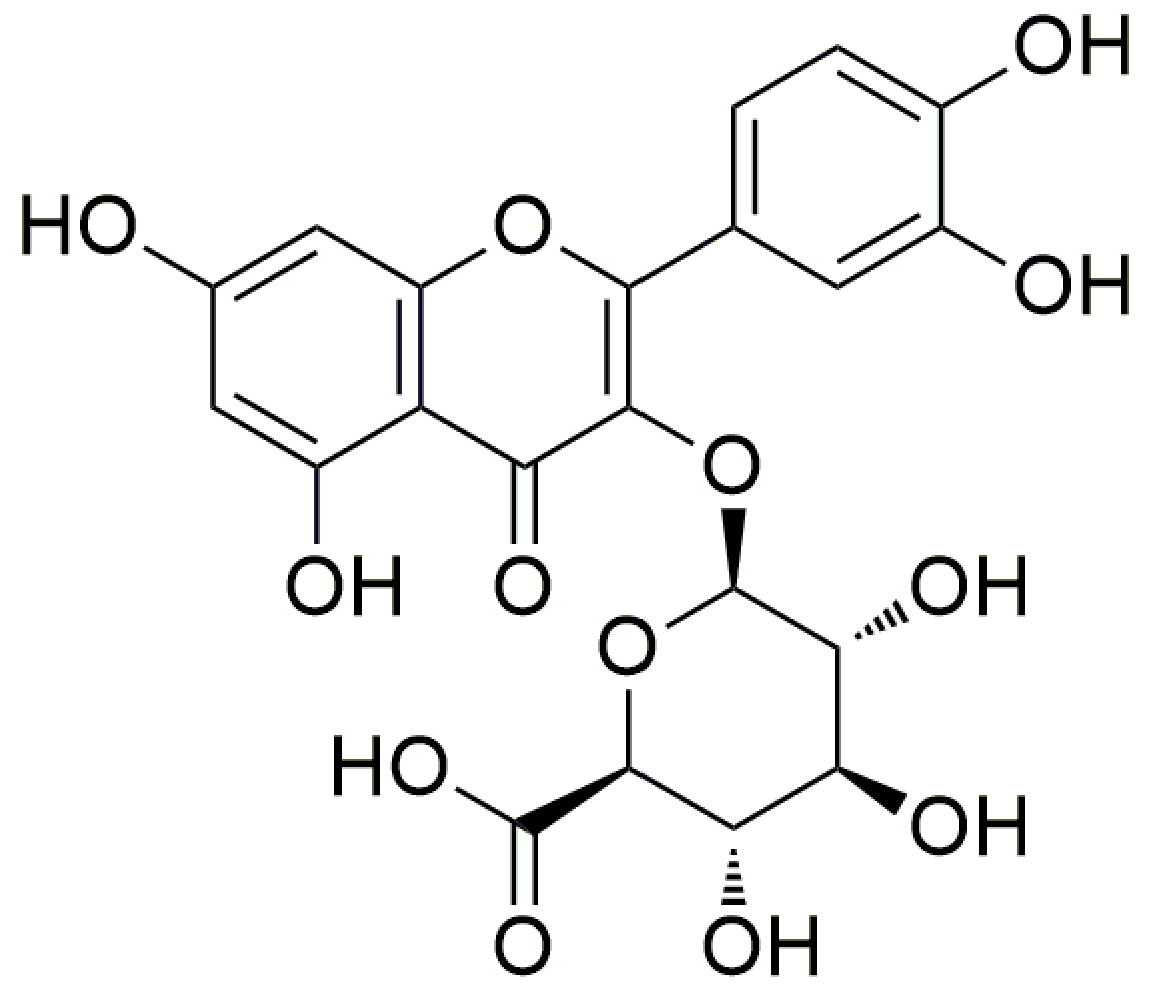

Supplement: Supplemental Information 4 [file peerj-07-6665-s004.zip › Supplemental_Data_S1_a/Quercetin-3-O-a┬-D-glucuronide (QG).png]

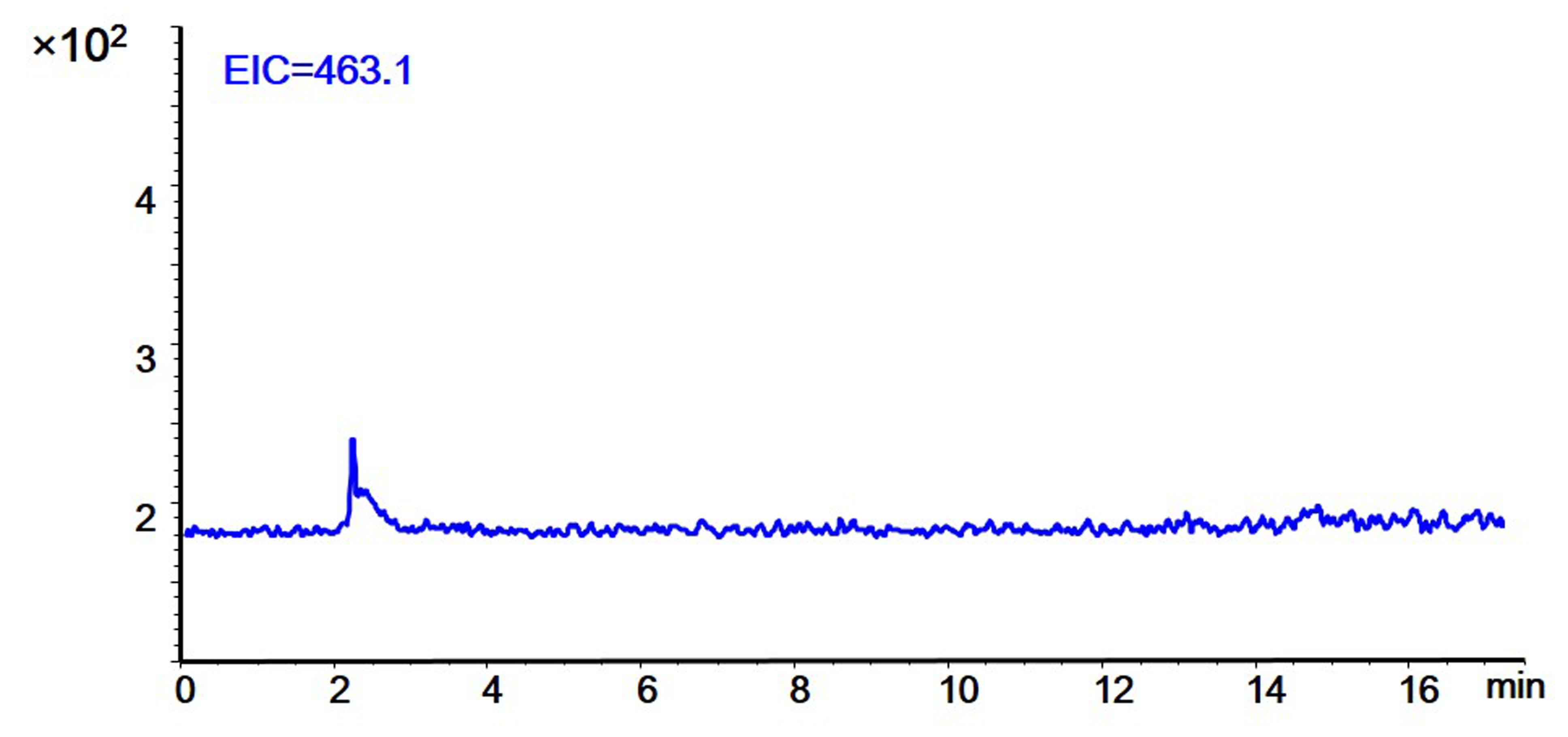

Supplement: Supplemental Information 5 — Raw data exported from the Agilent ChemStation Software applied for preparation for Fig. 2 for the selected ion monitoring chromatograms of puerarin (IS), quercetin (Qr), isoquercitrin (IQ), and quercetin-3-O-β-D-glucuronide (QG). [file peerj-07-6665-s005.zip › Supplemental_Data_S1_b/blank-IQ.png]

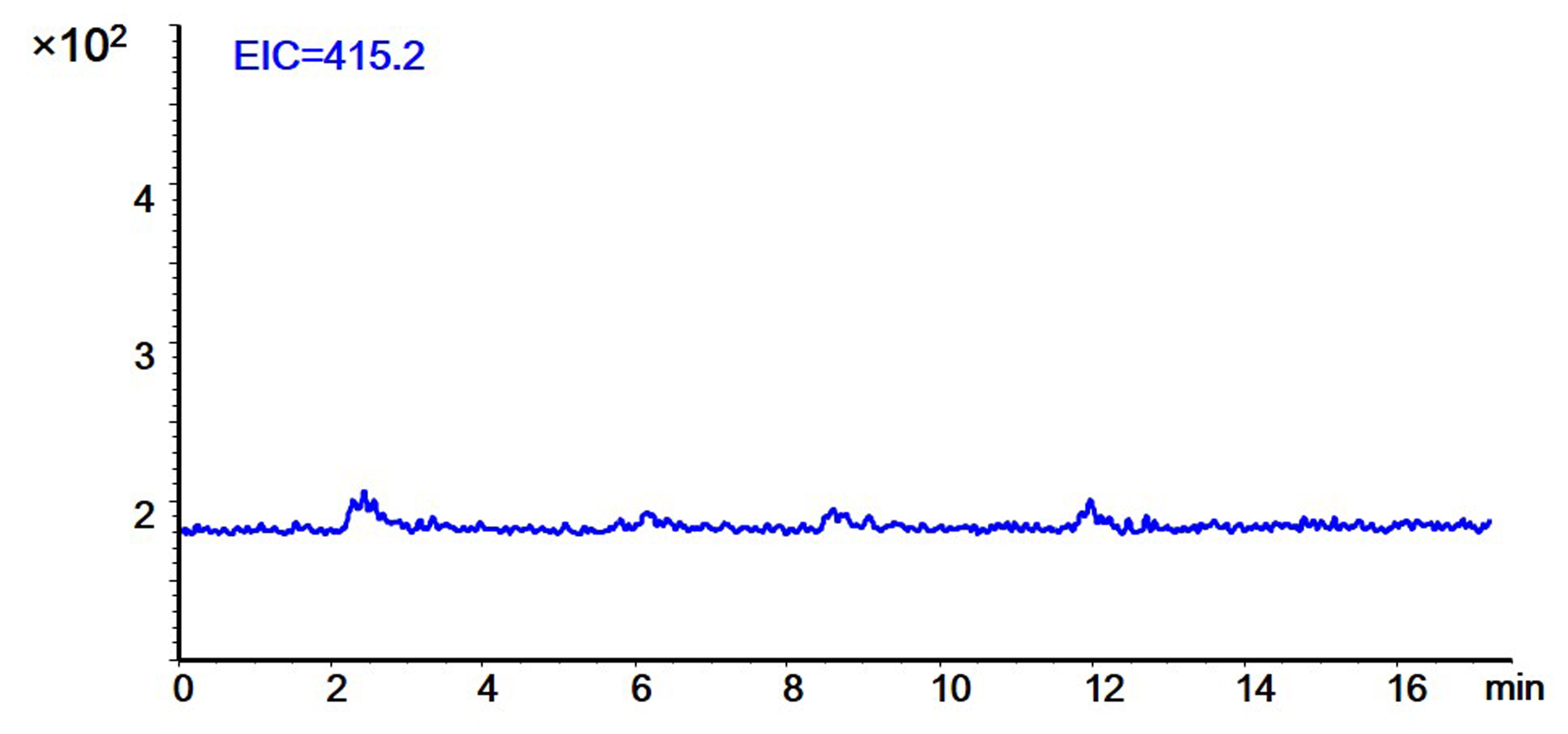

Supplement: Supplemental Information 5 — Raw data exported from the Agilent ChemStation Software applied for preparation for Fig. 2 for the selected ion monitoring chromatograms of puerarin (IS), quercetin (Qr), isoquercitrin (IQ), and quercetin-3-O-β-D-glucuronide (QG). [file peerj-07-6665-s005.zip › Supplemental_Data_S1_b/blank-IS.png]

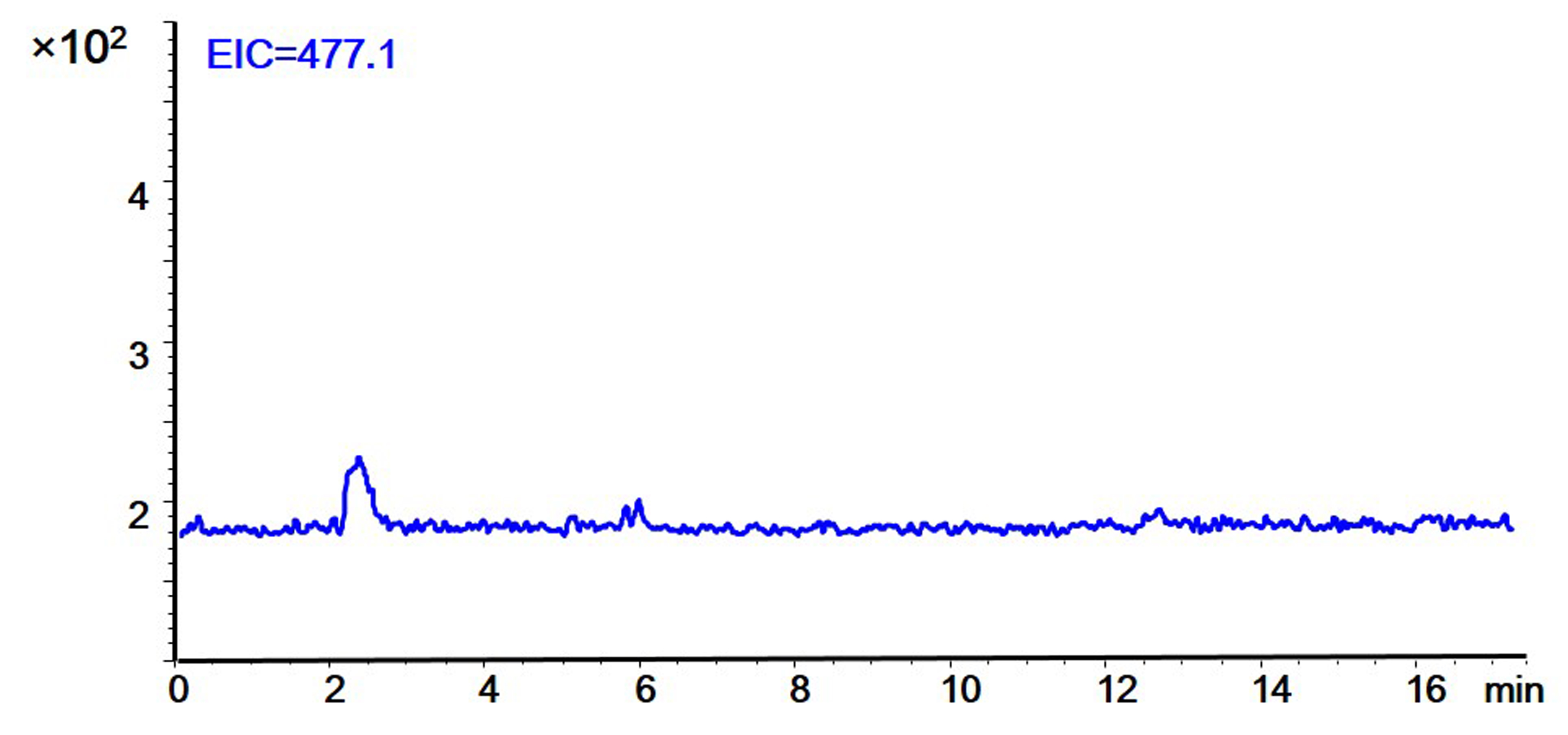

Supplement: Supplemental Information 5 — Raw data exported from the Agilent ChemStation Software applied for preparation for Fig. 2 for the selected ion monitoring chromatograms of puerarin (IS), quercetin (Qr), isoquercitrin (IQ), and quercetin-3-O-β-D-glucuronide (QG). [file peerj-07-6665-s005.zip › Supplemental_Data_S1_b/blank-QG.png]

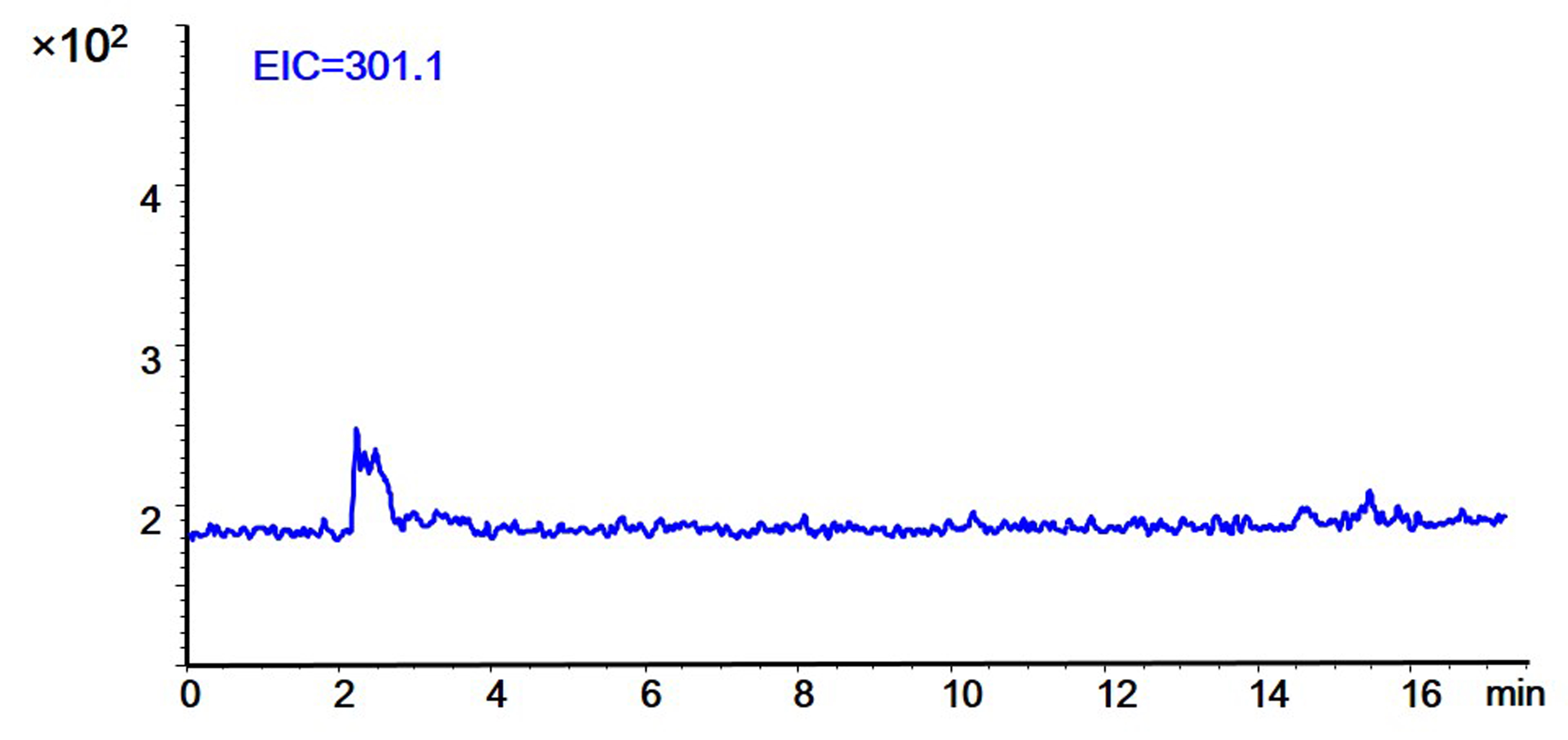

Supplement: Supplemental Information 5 — Raw data exported from the Agilent ChemStation Software applied for preparation for Fig. 2 for the selected ion monitoring chromatograms of puerarin (IS), quercetin (Qr), isoquercitrin (IQ), and quercetin-3-O-β-D-glucuronide (QG). [file peerj-07-6665-s005.zip › Supplemental_Data_S1_b/blank-Qr.png]

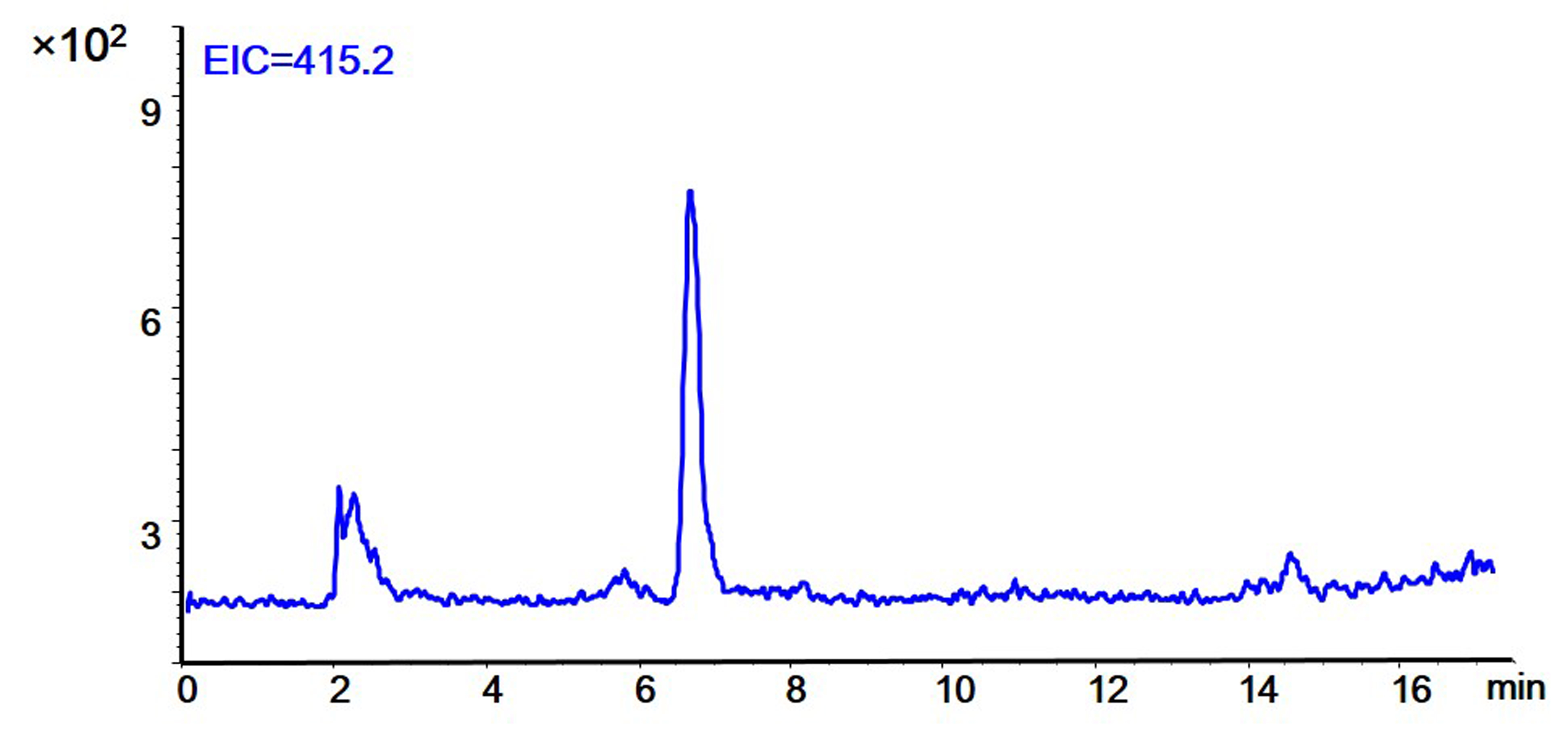

Supplement: Supplemental Information 5 — Raw data exported from the Agilent ChemStation Software applied for preparation for Fig. 2 for the selected ion monitoring chromatograms of puerarin (IS), quercetin (Qr), isoquercitrin (IQ), and quercetin-3-O-β-D-glucuronide (QG). [file peerj-07-6665-s005.zip › Supplemental_Data_S1_b/sample+IS.png]

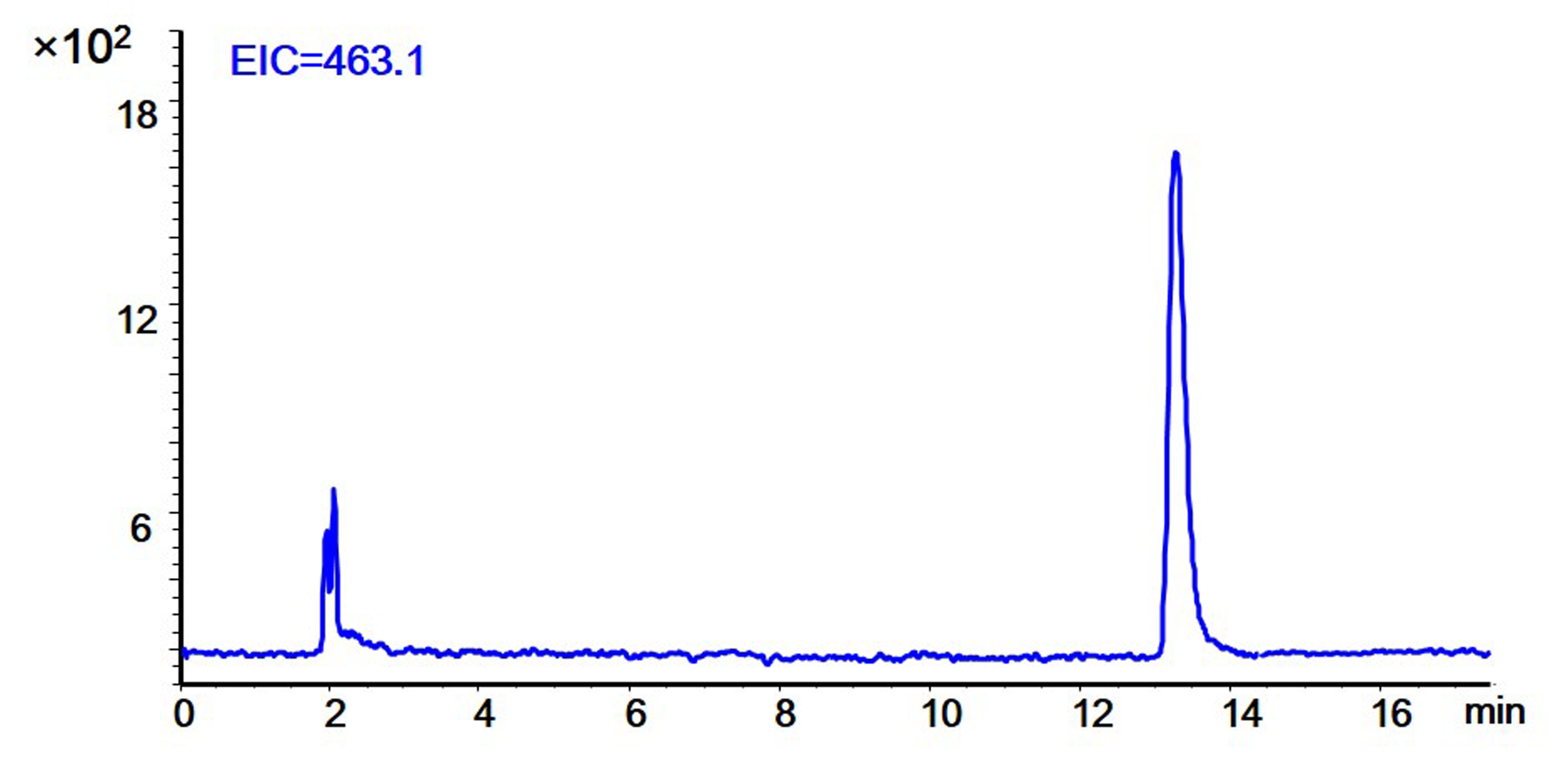

Supplement: Supplemental Information 5 — Raw data exported from the Agilent ChemStation Software applied for preparation for Fig. 2 for the selected ion monitoring chromatograms of puerarin (IS), quercetin (Qr), isoquercitrin (IQ), and quercetin-3-O-β-D-glucuronide (QG). [file peerj-07-6665-s005.zip › Supplemental_Data_S1_b/sample-IQ.png]

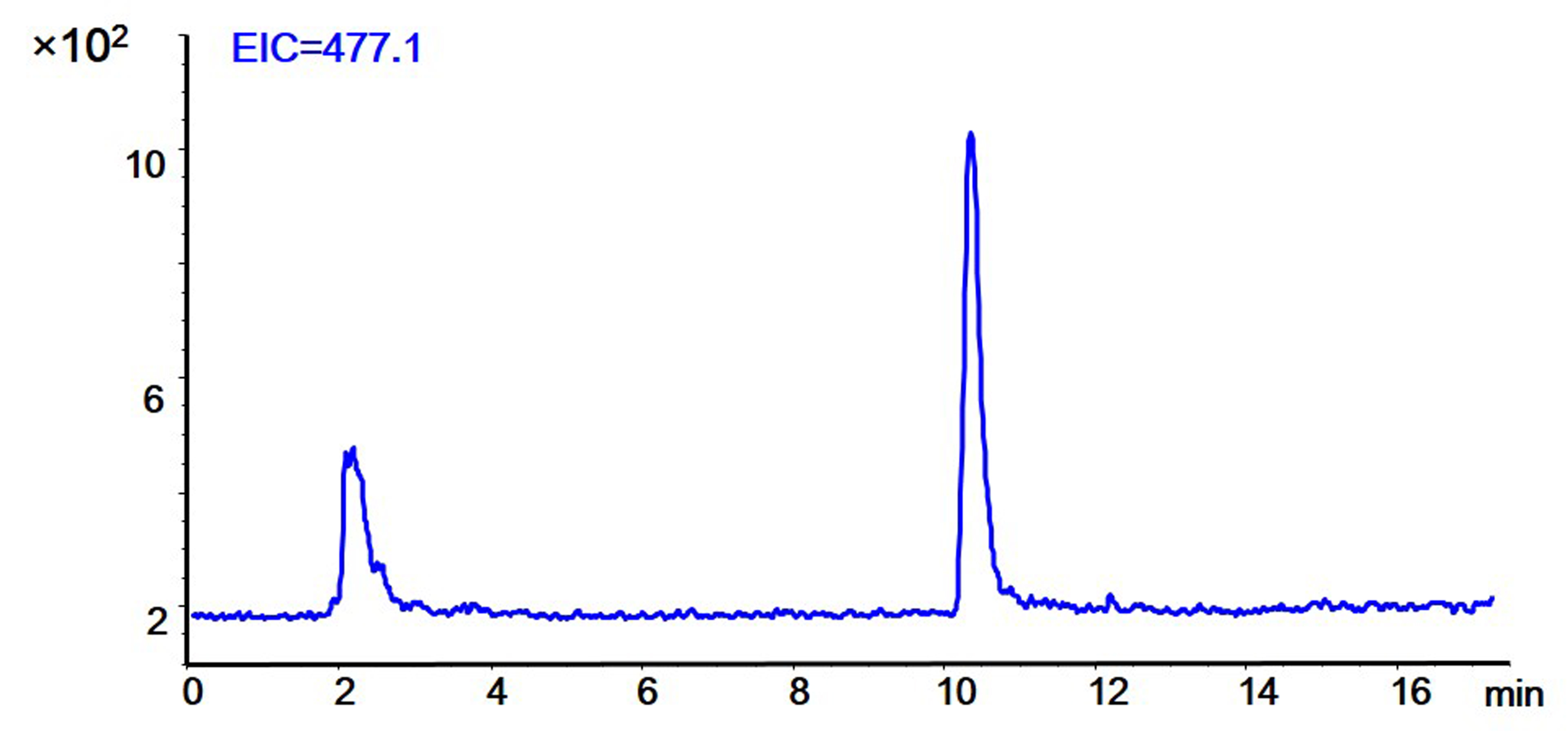

Supplement: Supplemental Information 5 — Raw data exported from the Agilent ChemStation Software applied for preparation for Fig. 2 for the selected ion monitoring chromatograms of puerarin (IS), quercetin (Qr), isoquercitrin (IQ), and quercetin-3-O-β-D-glucuronide (QG). [file peerj-07-6665-s005.zip › Supplemental_Data_S1_b/sample-QG.png]

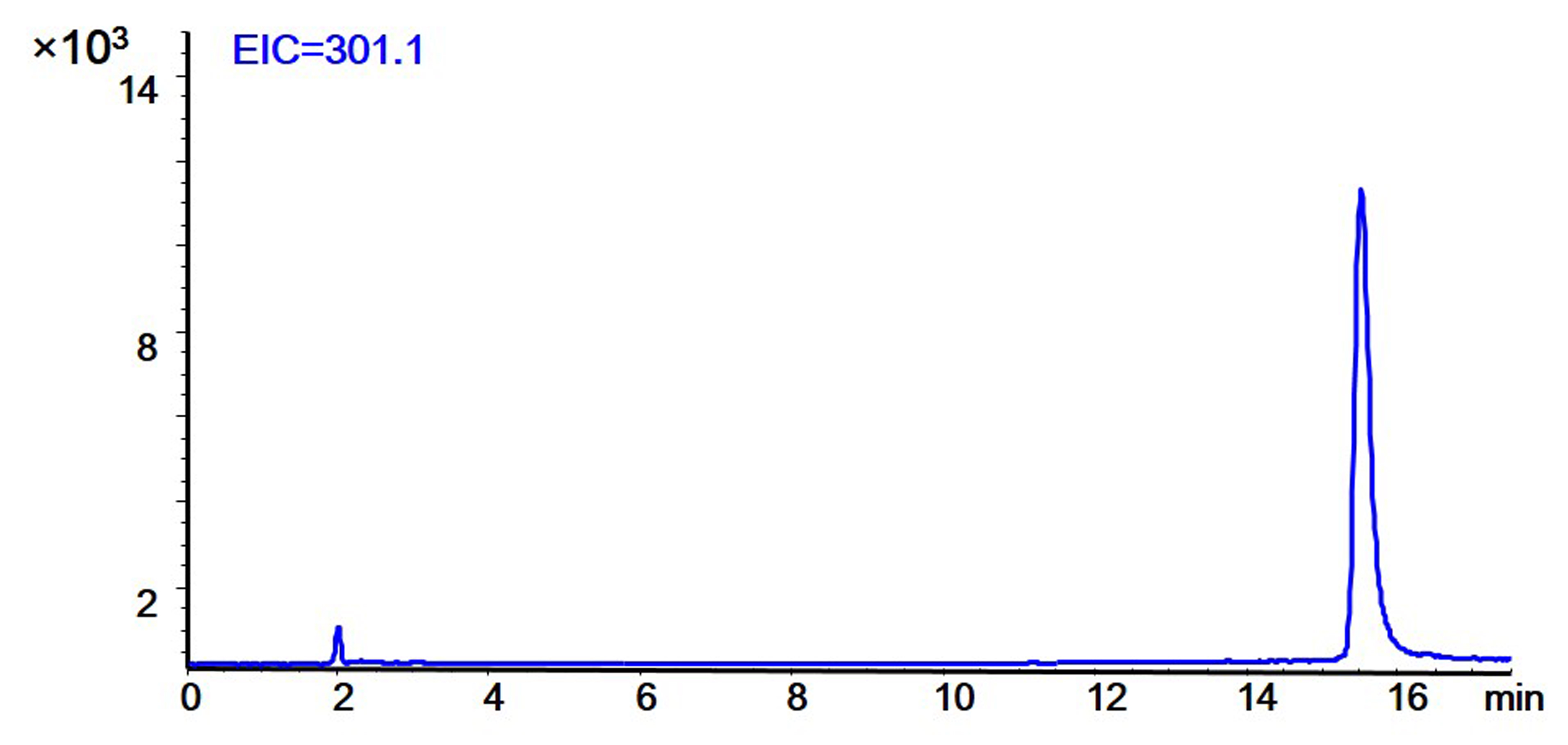

Supplement: Supplemental Information 5 — Raw data exported from the Agilent ChemStation Software applied for preparation for Fig. 2 for the selected ion monitoring chromatograms of puerarin (IS), quercetin (Qr), isoquercitrin (IQ), and quercetin-3-O-β-D-glucuronide (QG). [file peerj-07-6665-s005.zip › Supplemental_Data_S1_b/sample-Qr.png]

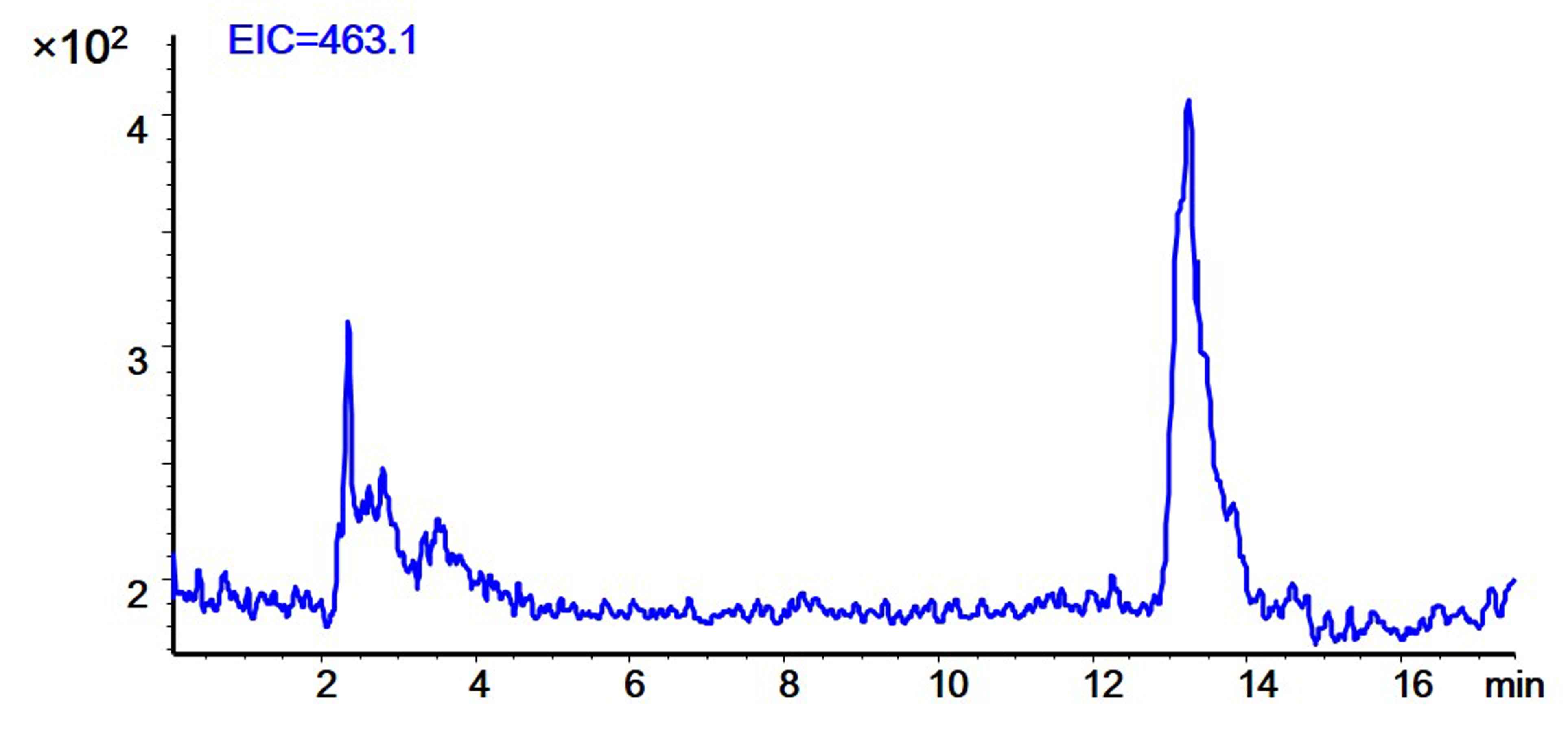

Supplement: Supplemental Information 5 — Raw data exported from the Agilent ChemStation Software applied for preparation for Fig. 2 for the selected ion monitoring chromatograms of puerarin (IS), quercetin (Qr), isoquercitrin (IQ), and quercetin-3-O-β-D-glucuronide (QG). [file peerj-07-6665-s005.zip › Supplemental_Data_S1_b/spiked+IQ.png]

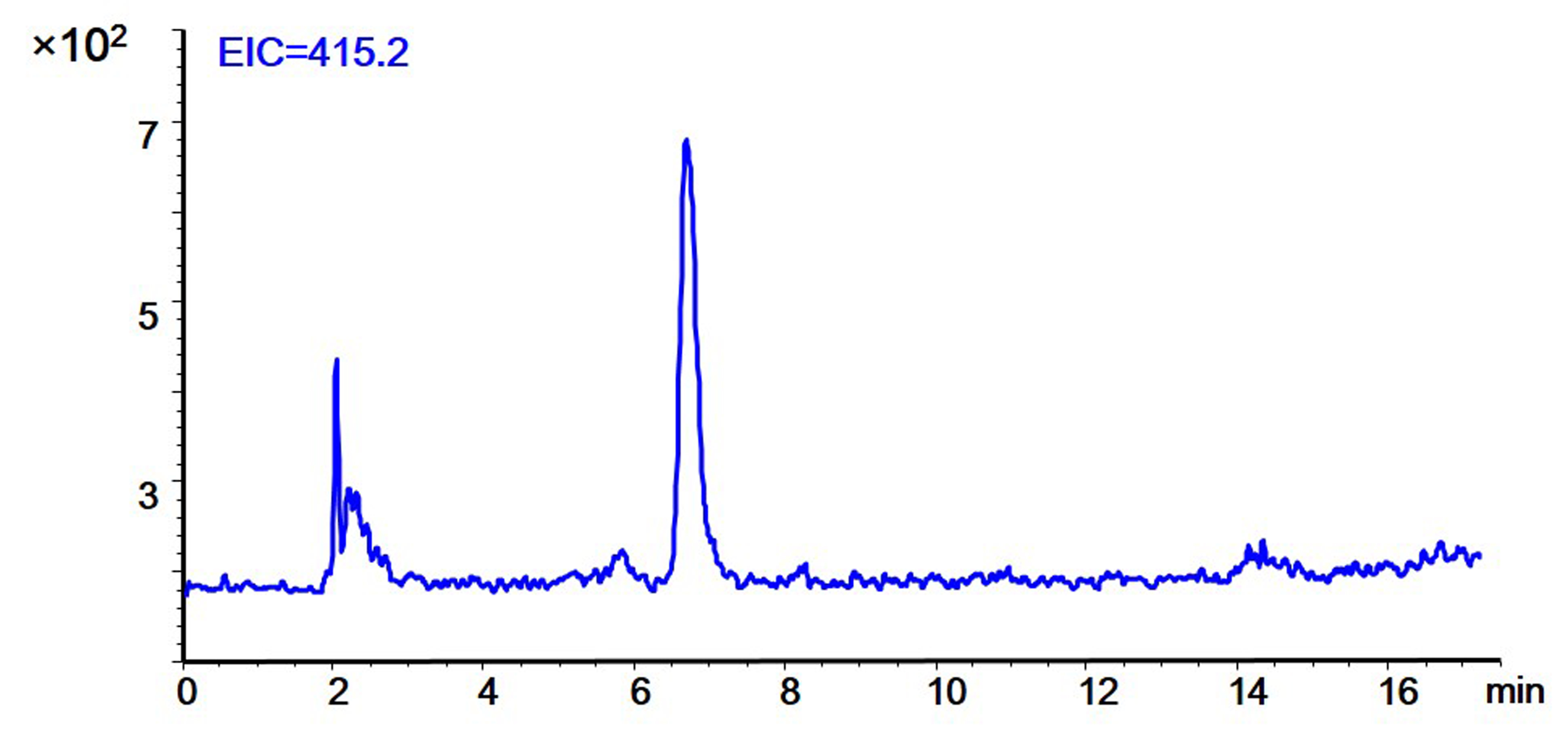

Supplement: Supplemental Information 5 — Raw data exported from the Agilent ChemStation Software applied for preparation for Fig. 2 for the selected ion monitoring chromatograms of puerarin (IS), quercetin (Qr), isoquercitrin (IQ), and quercetin-3-O-β-D-glucuronide (QG). [file peerj-07-6665-s005.zip › Supplemental_Data_S1_b/spiked+IS.png]

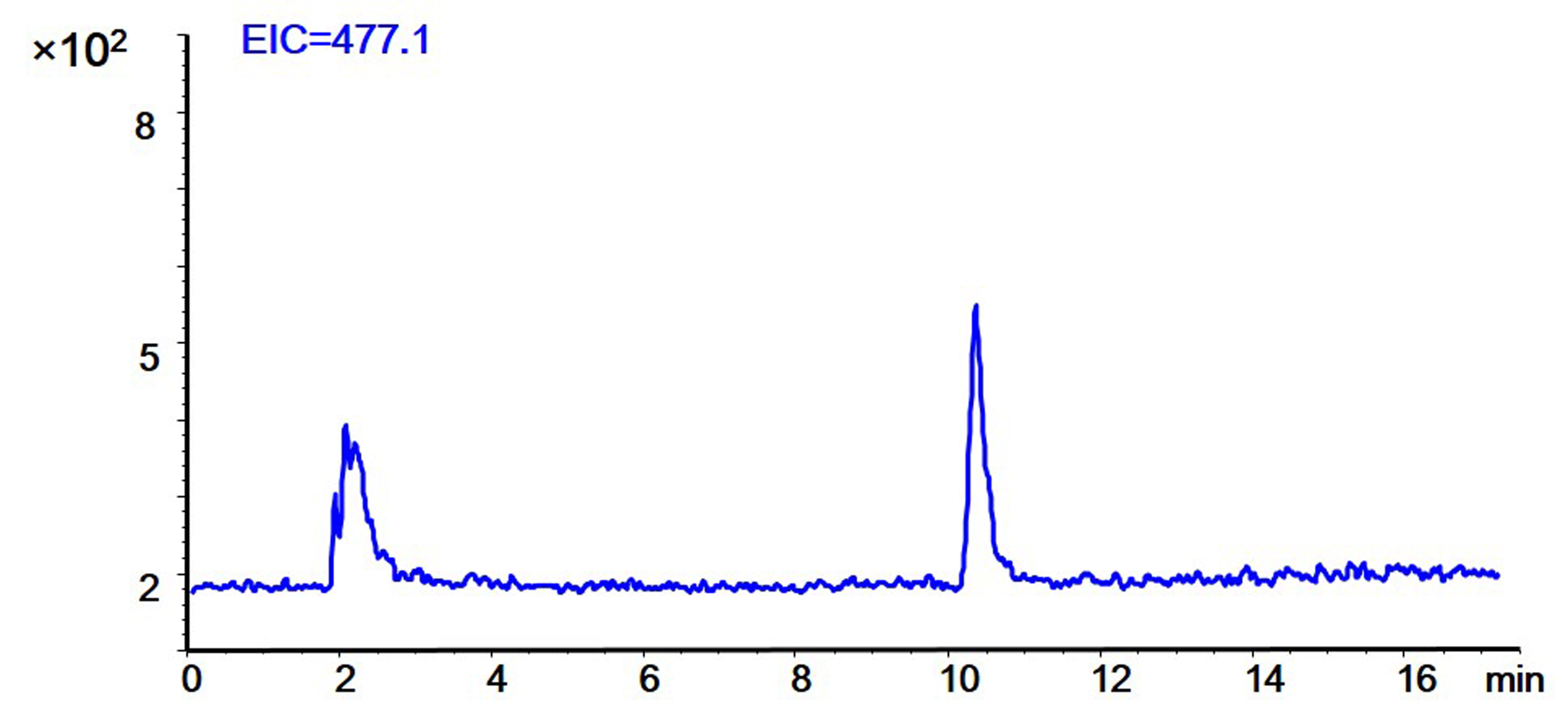

Supplement: Supplemental Information 5 — Raw data exported from the Agilent ChemStation Software applied for preparation for Fig. 2 for the selected ion monitoring chromatograms of puerarin (IS), quercetin (Qr), isoquercitrin (IQ), and quercetin-3-O-β-D-glucuronide (QG). [file peerj-07-6665-s005.zip › Supplemental_Data_S1_b/spiked+QG.png]

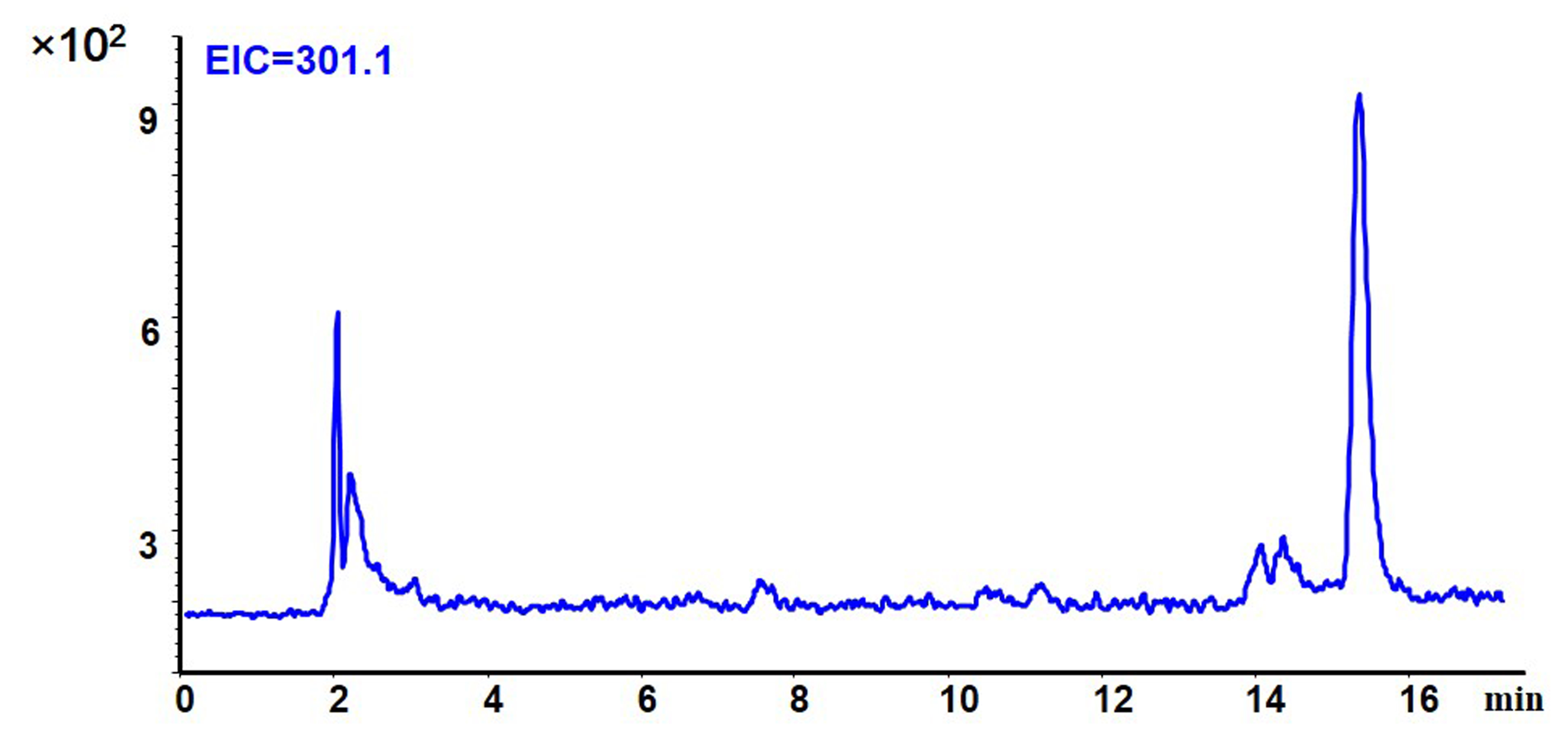

Supplement: Supplemental Information 5 — Raw data exported from the Agilent ChemStation Software applied for preparation for Fig. 2 for the selected ion monitoring chromatograms of puerarin (IS), quercetin (Qr), isoquercitrin (IQ), and quercetin-3-O-β-D-glucuronide (QG). [file peerj-07-6665-s005.zip › Supplemental_Data_S1_b/spiked+Qr.png]

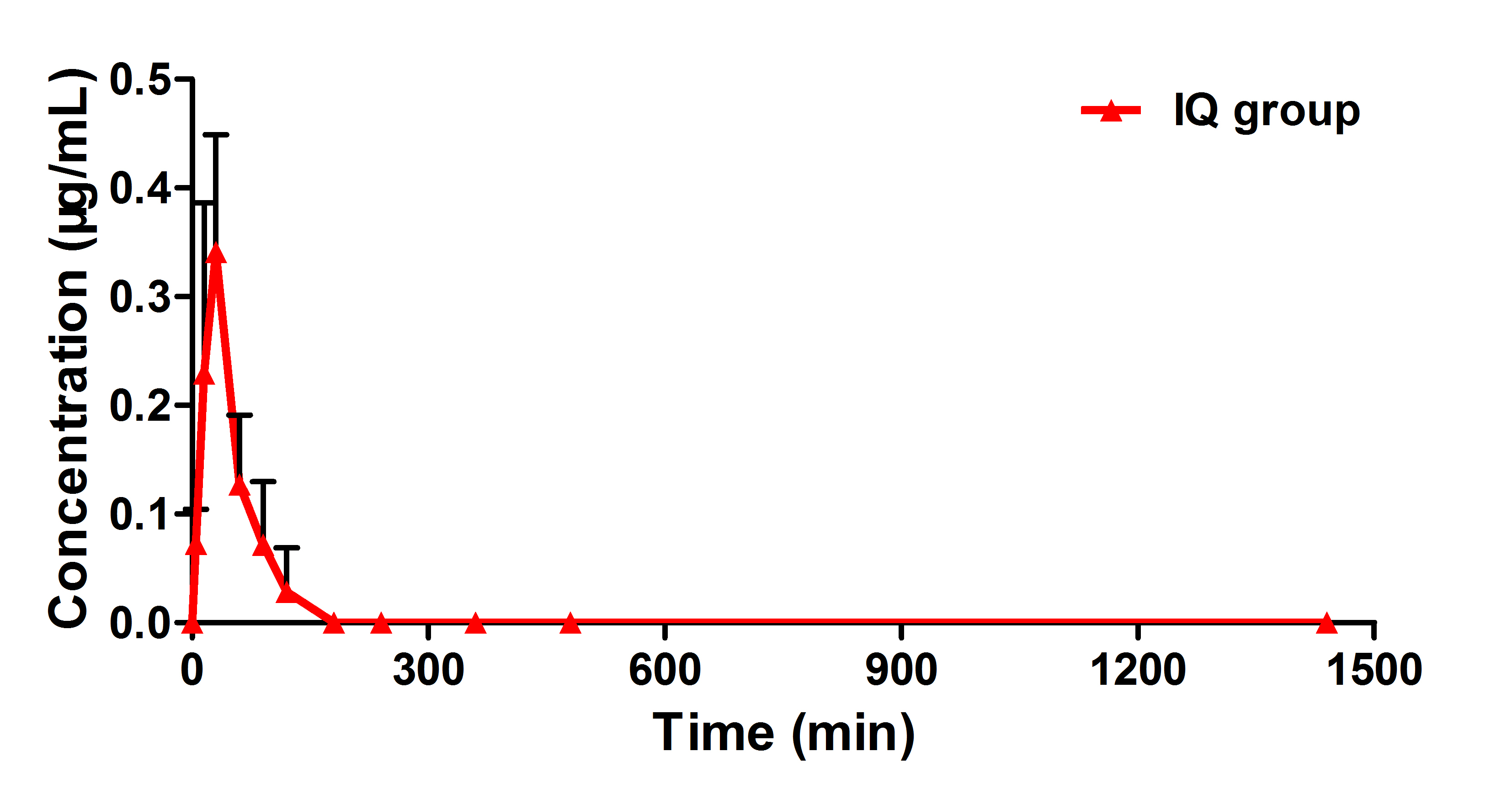

Supplement: Supplemental Information 6 [file peerj-07-6665-s006.zip › Supplemental_Data_S1_c/Isoquercitrin (IQ).png]

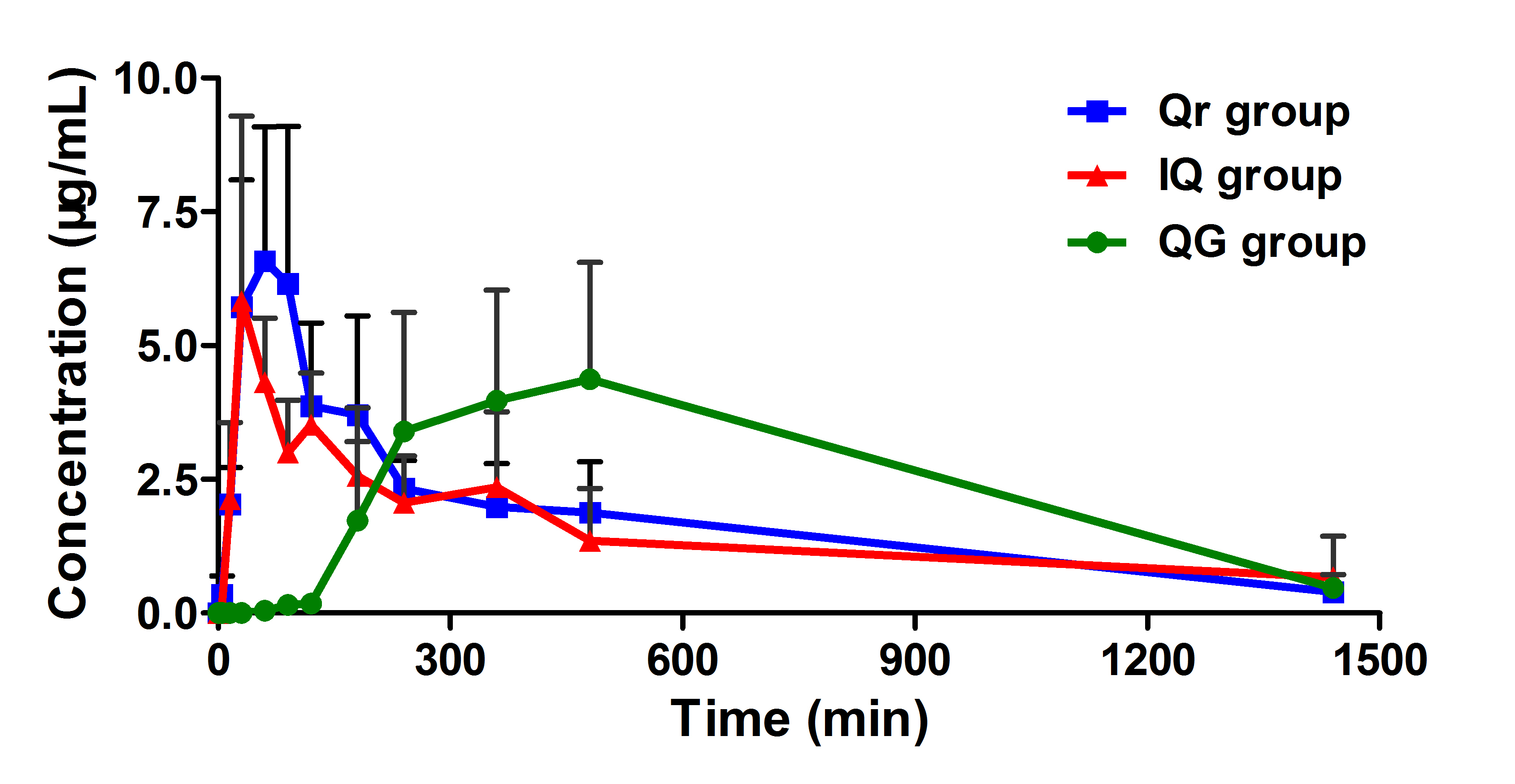

Supplement: Supplemental Information 6 [file peerj-07-6665-s006.zip › Supplemental_Data_S1_c/Quercetin (Qr).png]

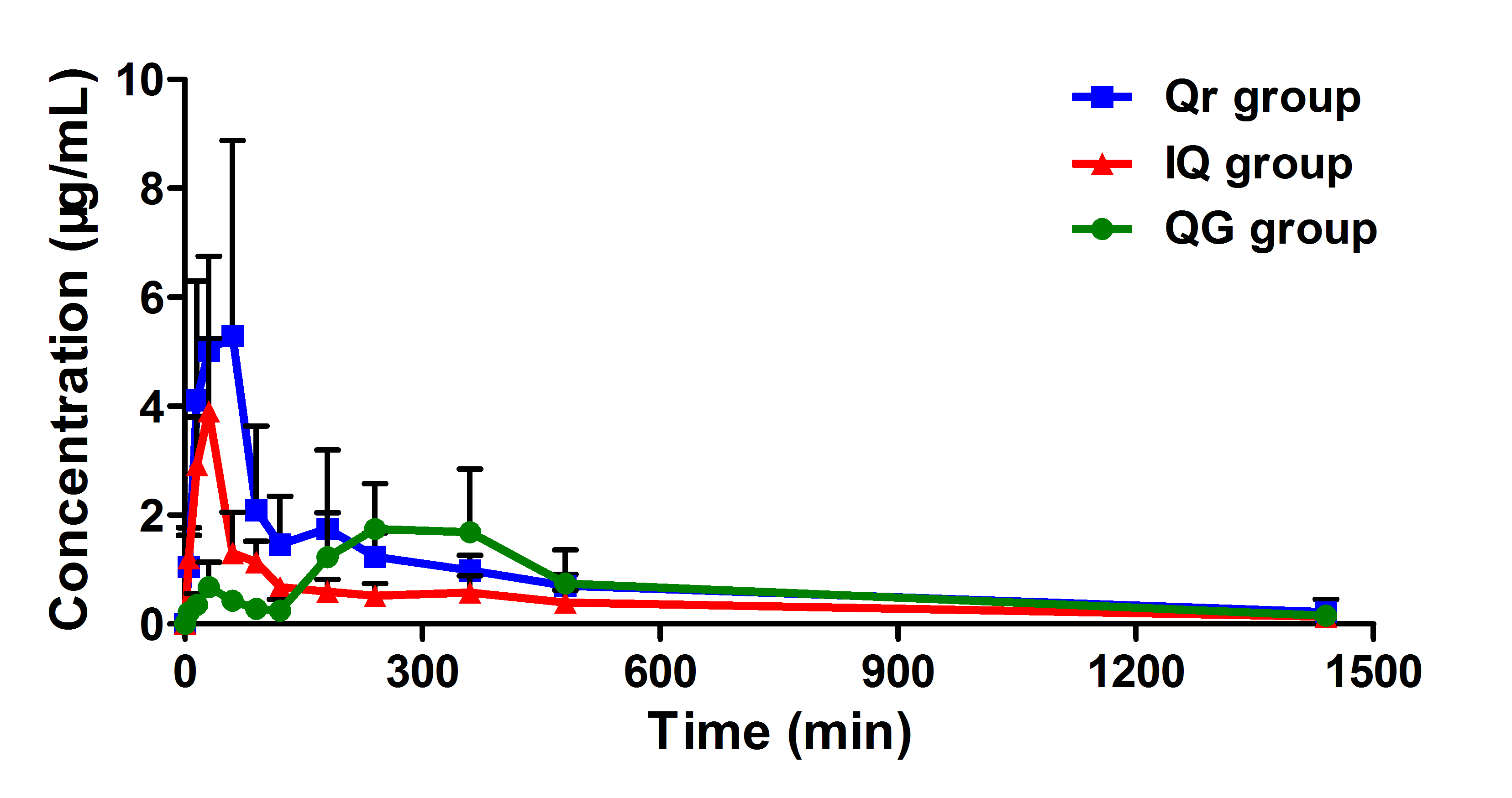

Supplement: Supplemental Information 6 [file peerj-07-6665-s006.zip › Supplemental_Data_S1_c/Quercetin-3-O-a┬-D-glucuronide (QG).png]
