# Supplementary material for: Pharmacokinetic comparison of quercetin, isoquercitrin, and quercetin-3-O-β-D-glucuronide in rats by HPLC-MS
Source: PeerJ. 2019 Mar 26;7:e6665. doi: 10.7717/peerj.6665 (PMC6440464; doi:10.7717/peerj.6665)
Supplement: Supplemental Information 7 [file peerj-07-6665-s007.zip › Supplemental_Data_S1_d/Standard curve calculation steps.pptx]

## Slide 1
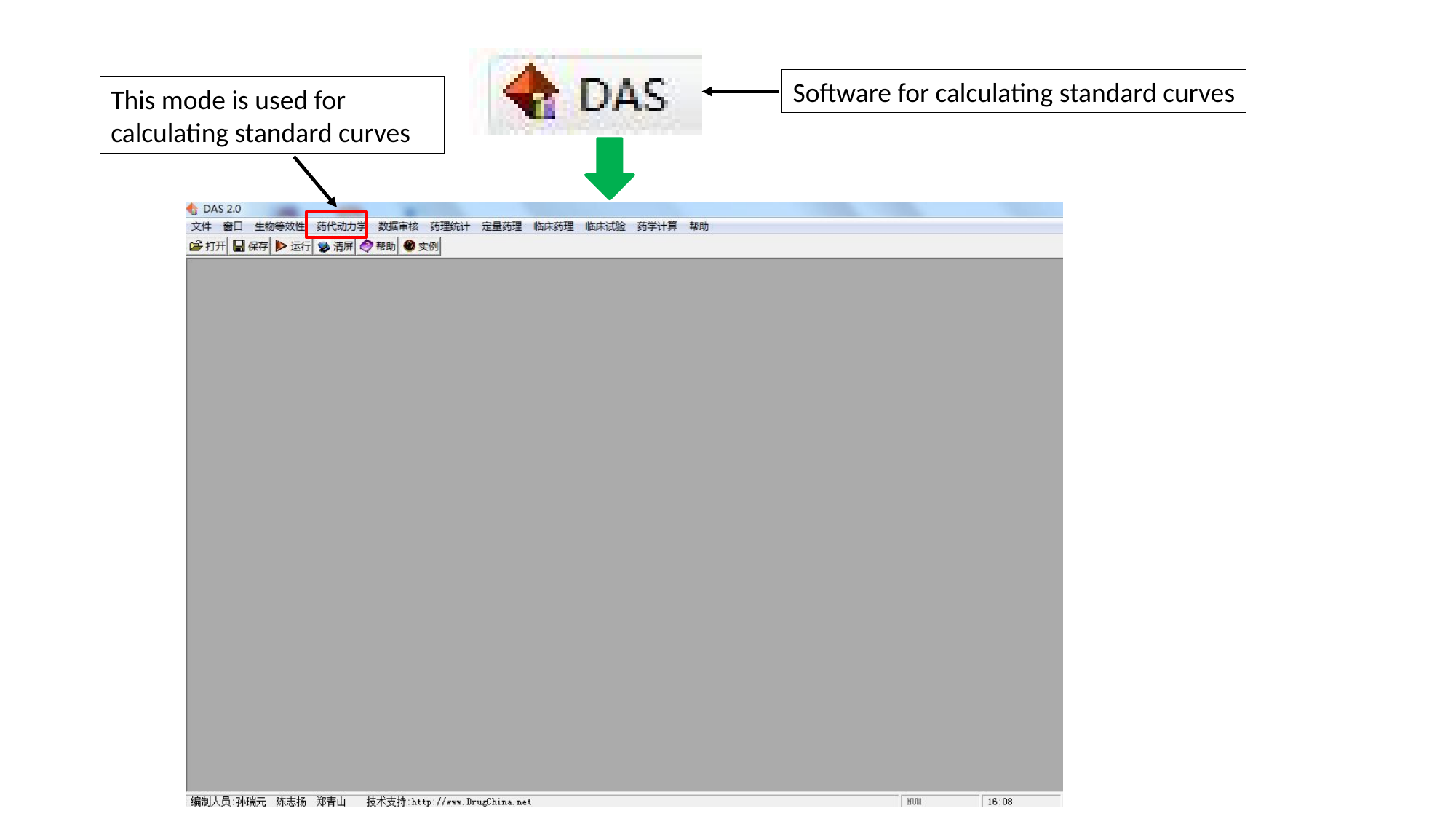

Software for calculating standard curves
This mode is used for calculating standard curves

## Slide 2
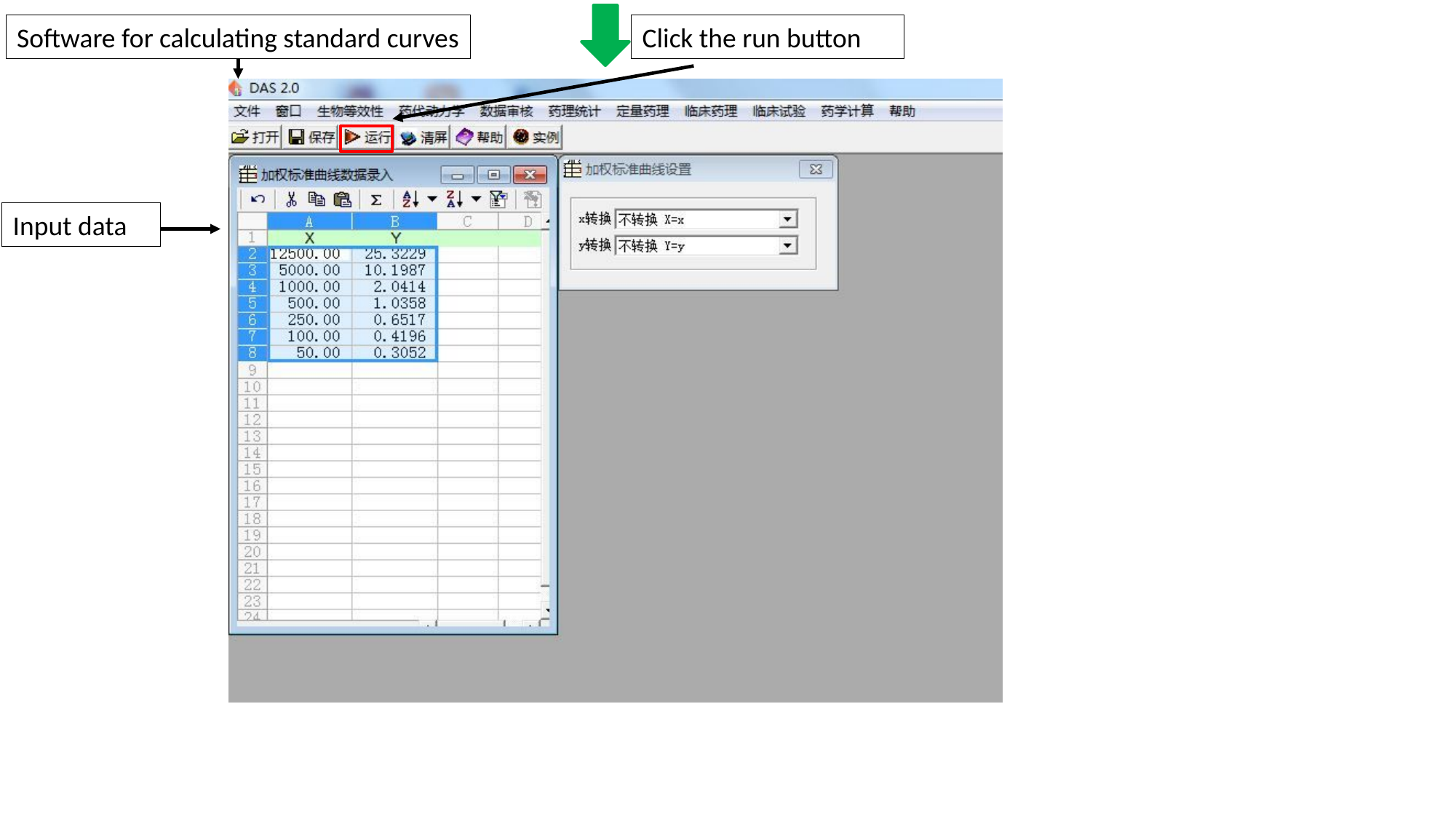

Software for calculating standard curves
Click the run button
Input data

## Slide 3
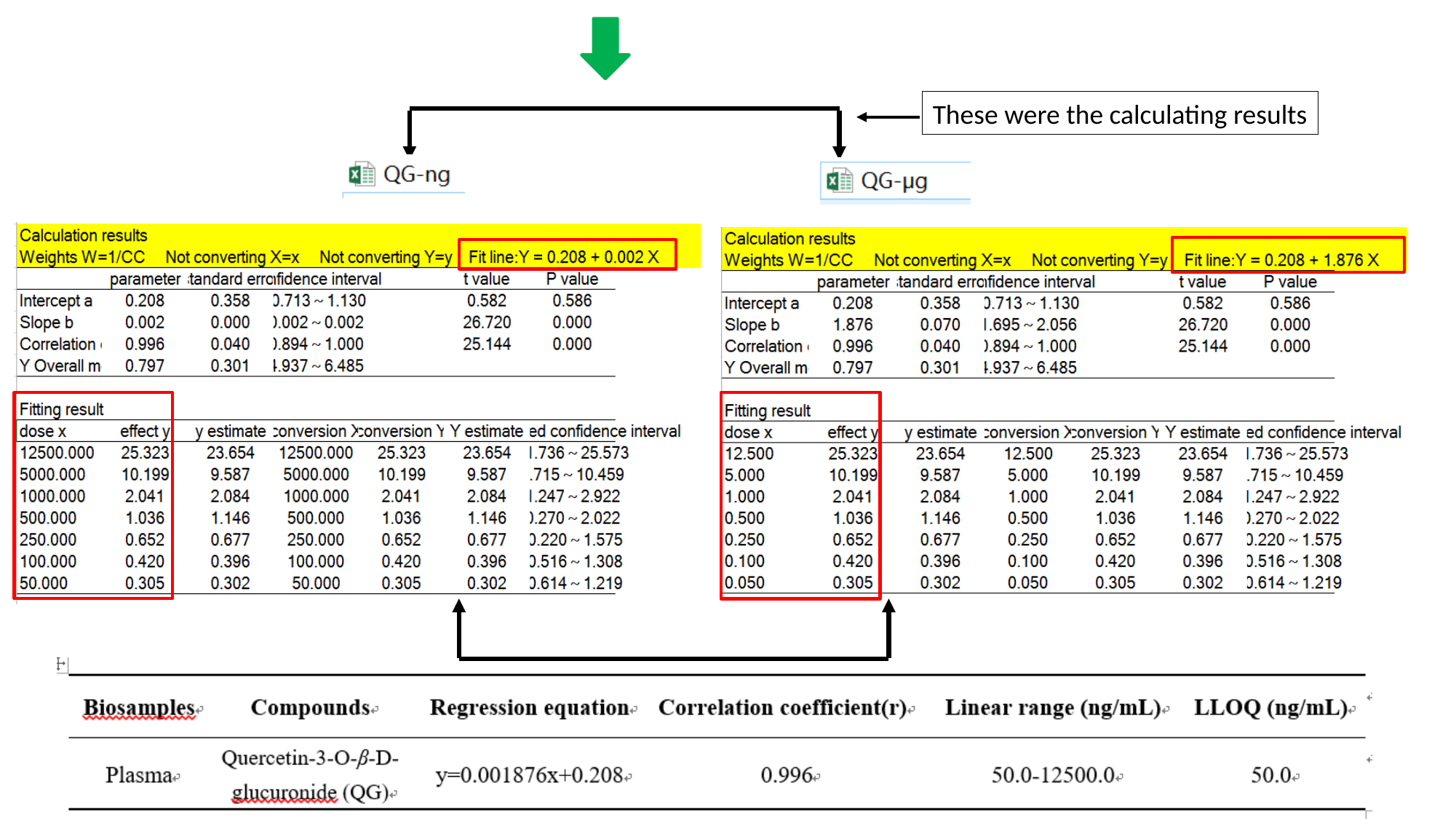

These were the calculating results
