# Supplementary material for: Pharmacokinetic comparison of quercetin, isoquercitrin, and quercetin-3-O-β-D-glucuronide in rats by HPLC-MS
Source: PeerJ. 2019 Mar 26;7:e6665. doi: 10.7717/peerj.6665 (PMC6440464; doi:10.7717/peerj.6665)
Supplement: Supplemental Information 10 [file peerj-07-6665-s010.zip › Supplemental_Data_S1_g/Calculation method description.pptx]

## Slide 1
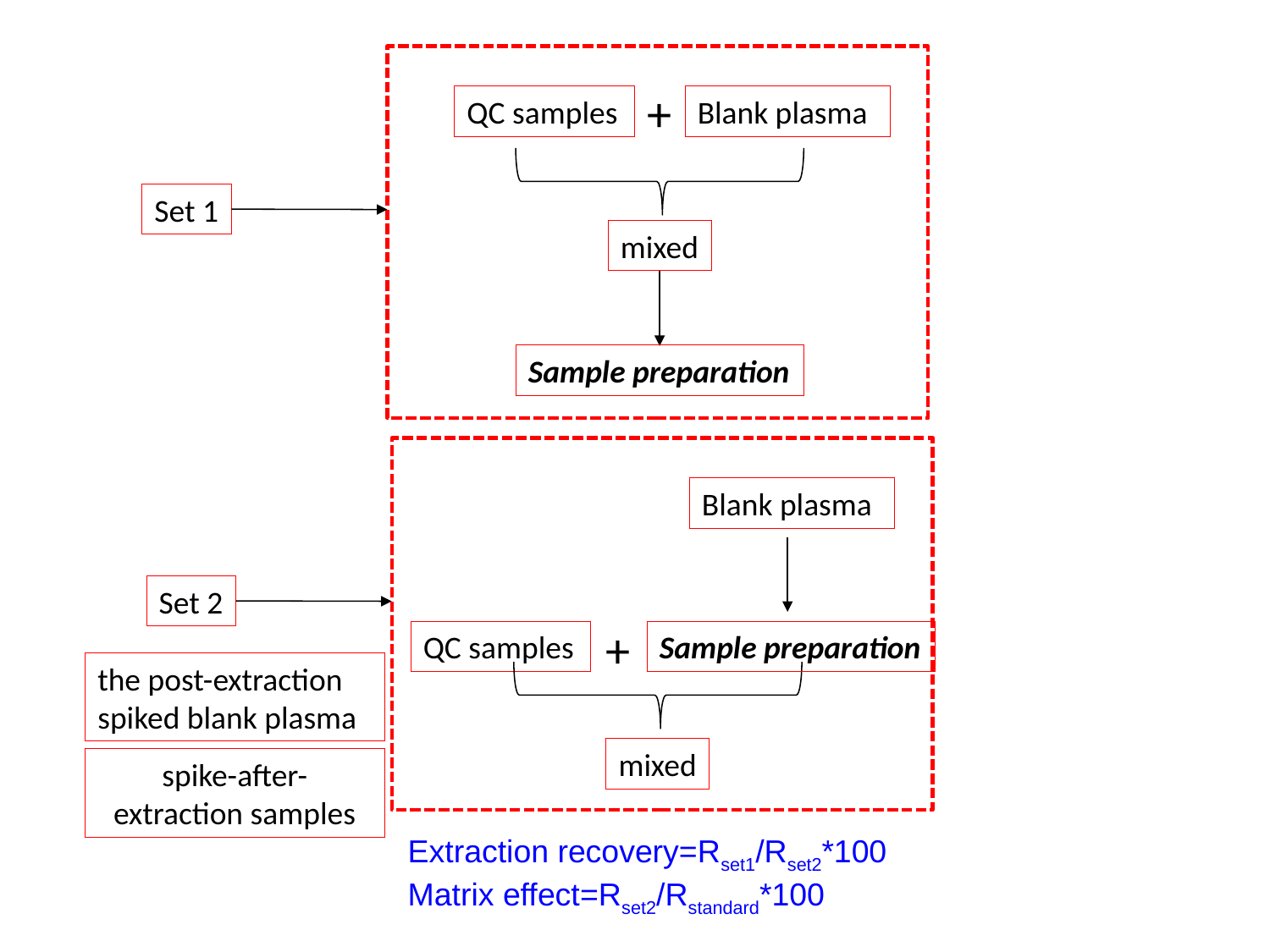

+
QC samples
Blank plasma
Set 1
mixed
Sample preparation
Blank plasma
Set 2
+
QC samples
Sample preparation
mixed
the post-extraction spiked blank plasma
spike-after-extraction samples
Extraction recovery=Rset1/Rset2*100
Matrix effect=Rset2/Rstandard*100
